# A Curated Database of Rodent Uterotrophic Bioactivity

**DOI:** 10.1289/ehp.1510183

**Published:** 2015-10-02

**Authors:** Nicole C. Kleinstreuer, Patricia C. Ceger, David G. Allen, Judy Strickland, Xiaoqing Chang, Jonathan T. Hamm, Warren M. Casey

**Affiliations:** 1 Integrated Laboratory Systems, in support of the National Toxicology Program Interagency Center for Evaluation of Alternative Toxicological Methods (NICEATM), Research Triangle Park, North Carolina, USA; 2 NICEATM, Division of the National Toxicology Program, National Institute of Environmental Health Sciences, National Institutes of Health, Department of Health and Human Services, Research Triangle Park, North Carolina, USA

## Abstract

**Background:**

Novel in vitro methods are being developed to identify chemicals that may interfere with estrogen receptor (ER) signaling, but the results are difficult to put into biological context because of reliance on reference chemicals established using results from other in vitro assays and because of the lack of high-quality in vivo reference data. The Organisation for Economic Co-operation and Development (OECD)-validated rodent uterotrophic bioassay is considered the “gold standard” for identifying potential ER agonists.

**Objectives:**

We performed a comprehensive literature review to identify and evaluate data from uterotrophic studies and to analyze study variability.

**Methods:**

We reviewed 670 articles with results from 2,615 uterotrophic bioassays using 235 unique chemicals. Study descriptors, such as species/strain, route of administration, dosing regimen, lowest effect level, and test outcome, were captured in a database of uterotrophic results. Studies were assessed for adherence to six criteria that were based on uterotrophic regulatory test guidelines. Studies meeting all six criteria (458 bioassays on 118 unique chemicals) were considered guideline-like (GL) and were subsequently analyzed.

**Results:**

The immature rat model was used for 76% of the GL studies. Active outcomes were more prevalent across rat models (74% active) than across mouse models (36% active). Of the 70 chemicals with at least two GL studies, 18 (26%) had discordant outcomes and were classified as both active and inactive. Many discordant results were attributable to differences in study design (e.g., injection vs. oral dosing).

**Conclusions:**

This uterotrophic database provides a valuable resource for understanding in vivo outcome variability and for evaluating the performance of in vitro assays that measure estrogenic activity.

**Citation:**

Kleinstreuer NC, Ceger PC, Allen DG, Strickland J, Chang X, Hamm JT, Casey WM. 2016. A curated database of rodent uterotrophic bioactivity. Environ Health Perspect 124:556–562; http://dx.doi.org/10.1289/ehp.1510183

## Introduction

Understanding the impact of endocrine bioactive chemicals on human health and the environment is a high priority for U.S. and international agencies. The large number of untested chemicals in commerce (> 80,000) necessitates the use of high-throughput screening (HTS) programs such as the U.S. Environmental Protection Agency (EPA) ToxCast^TM^ initiative and the Tox21 U.S. federal partnership to quickly identify potential endocrine disruptors and to help characterize any hazards they may pose (Dix et al. 2007; Judson et al. 2010; Kavlock et al. 2012; Tice et al. 2013; U.S. EPA 2011a, 2012). Furthermore, there is growing societal pressure to avoid animal testing and to develop alternative approaches that replace, reduce, or refine the use of animals in toxicity testing [Hartung 2009; Interagency Coordinating Committee on the Validation of Alternative Methods (ICCVAM) Authorization Act of 2000].

To determine the usefulness and limitations of a novel alternative method for identifying endocrine activity and to show that it is fit for its intended purpose, the method must be evaluated against a set of chemicals that have demonstrated activity and well-defined properties (potency and efficacy) against the target nuclear receptor and the subsequent biological pathway. At the present time, reference chemicals used to validate *in vitro* assays aimed at detecting potential endocrine disruptors (estrogen, androgen, and thyroid receptors) are selected based only on their activity in other *in vitro* assays, a circular validation paradigm that arose because of the lack of sufficient *in vivo* data [ICCVAM et al. 2011; Organisaton for Economic Co-operation and Development (OECD) 2012]. To facilitate work that will better elucidate and characterize the relationship between the *in vitro* and *in vivo* estrogen bioactivity of chemicals, the National Toxicology Program Interagency Center for Evaluation of Alternative Toxicological Methods (NICEATM) developed a curated database of high-quality *in vivo* rodent uterotrophic bioassay data extracted from published studies (http://ntp.niehs.nih.gov/pubhealth/evalatm/tox21-support/endocrine-disruptors/edhts.html).

The uterotrophic bioassay [Test Guideline (TG) 440] was validated by the OECD as a short-term screening test to evaluate the ability of a substance to elicit estrogenic activity (Kanno et al. 2001, 2003; OECD 2004; Owens and Koëter 2003). This bioassay is one of the 11 Tier 1 screening assays in the U.S. EPA’s endocrine-disruptor screening program (EDSP) and is considered the “gold standard” bioassay screen for identifying estrogen receptor (ER) agonists (U.S. EPA 2011b, 2012). The end point measured is an increase in uterine weight caused by ER-mediated water imbibition and cellular proliferation in the uterine tissue. According to the OECD (2004) and U.S. EPA (2011b) test guidelines for the uterotrophic assay, immature female rats or ovariectomized (OVX) adult female mice or rats can be used. Because immature and OVX animals do not produce endogenous estrogens, the uterus becomes sensitive to external estrogenic substances (Billon-Galés et al. 2011).

Herein, we describe a comprehensive database of quality-controlled *in vivo* uterotrophic studies. To create this database, we reviewed the current scientific literature as of December 2014 for studies that measured uterine weight changes in immature rats or OVX rats or mice, identified relevant assay parameters and end points, compiled the data into a single database, and analyzed the data for sources of variability. Our analysis revealed that certain protocol variations, specifically the use of rats versus mice and injection versus gavage dosing, were more likely to produce a positive response. This database was also used to assess the reproducibility of the uterotrophic bioassay and to provide a resource against which *in vitro* test method results for ER activity may be evaluated and from which predictive *in silico* models (Browne et al. 2015) may be built.

## Methods

**Figure 1:**
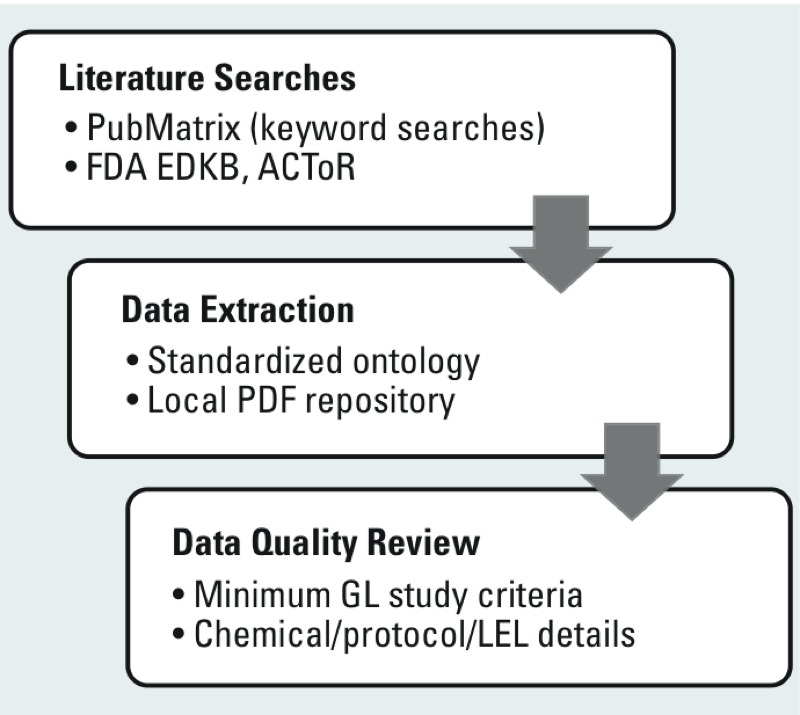
Flow diagram illustrating the curation of the uterotrophic database (UTDB) and identification of high-quality guideline-like (GL) studies. Abbreviations: ACToR, Aggregated Computational Toxicology Resource; FDA EDKB, U.S. Food and Drug Administration Endocrine Disruptor Knowledge Base; LEL, lowest effect level.

*Curation process.* NICEATM conducted a comprehensive literature search to identify uterotrophic studies for environmental chemicals. The ToxCast^TM^ Phase I/Phase II/E1K chemical library (1,812 substances, http://epa.gov/comptox/toxcast/data.html) was chosen as a starting point based on its relevance to the EDSP universe of chemicals and to facilitate future comparisons with results from the 18 HTS *in vitro* assays included in ToxCast^TM^ that map to the ER pathway (Judson et al. 2015; Rotroff et al. 2014). We performed semiautomated literature searches, reviewed relevant manuscripts, and recorded detailed study information for each chemical/study/protocol combination (Table 1) along with the reported bioactivity for the dose range tested. The literature search strategy and database development procedure are illustrated in Figure 1 and are detailed below.

**Table 1:** Study details (and examples) extracted from papers measuring uterine weight change.

Study information category	Examples^*a*^
Species	Rat, mouse
Strain	Sprague Dawley, Wistar, CD1, etc.
Study type	Immature, OVX, intact, etc.
Assay type	Organ weight
Assay target	Uterine weight
Route of administration	i.p. injection, s.c. injection, p.o., etc.
Age at first dose	PND 0, PND 18, adult, etc.
OVX status	OVX or NA
Age at OVX	PND 20, 5 weeks, NA, etc.
Dosing length	Single dose, 3 days, 3 weeks, etc.
Dosing frequency	Daily, twice daily, etc.
Number of doses	1, 2, 3, 4, etc.
Highest dose tested	500 mg/kg/day, etc.
Number of animals	3, 4, 5, 6, etc.
Positive control	Estradiol, ethinyl estradiol
Post-treatment necropsy time	24 hr, 1 day, etc.
LEL	0.1, 10, 100, etc.
LEL units	Milligrams per kilogram per day, milligrams per animal, etc.
Response observed	Increase, decrease, NA
Response value	1.5, 2; 150, 200; 0.01, 0.2; etc.
Response units	Fold change relative to control; percent increase; log relative potency; etc.
Abbreviations: i.p., intraperitoneal; LEL, lowest effect level; NA, not available; OVX, ovariectomized; PND, postnatal day; p.o., oral gavage; s.c., subcutaneous.^***a***^Examples for response units correspond to the types of response values collected.	

Searches were performed in a semiautomated fashion using the U.S. National Center for Biotechnology Information’s PubMatrix tool [http://pubmatrix.grc.nia.nih.gov/ (accessed August 2013–December 2014)]. PubMatrix is a web-based resource that provides a simple approach to rapidly and systematically comparing any list of (search) terms against any other list of (modifier) terms in PubMed. Lists of terms can include any keyword that may correspond to a Medical Subject Heading (MeSH) term, such as chemical names, genes, diseases, phenotypic observations, gene functions, or authors. Searches were performed in batches of 50 chemicals, using both chemical name and Chemical Abstracts Service Registry Number (CASRN) in the list of search terms. PubMatrix automatically identifies all chemical name synonyms in PubMed and includes these as alternative search terms. The modifier terms used to cross-reference and identify articles were “uterotrophic,” “uterotrophic assay,” and “uterine weight.” The modifier term “uterotropic” was also included as a common alternative to “uterotrophic.” The output of a PubMatrix search is a matrix table showing the frequency of co-occurrence between all pairwise comparisons between the two lists, with links out to the publications identified in the overlap space. We searched for additional studies in the U.S. Food and Drug Administration’s Endocrine Disruptor Knowledge Base (Ding et al. 2010) and the U.S. EPA’s Aggregated Computational Toxicology Resource (ACToR) database (Judson et al. 2008). Relevant publications were identified and downloaded for further manual curation, in which protocol information was entered into the NICEATM *in vivo* uterotrophic database (UTDB) so that each study could be evaluated for specifically defined quality control metrics as described below. Publications in languages other than English were included in the initial search results. These were evaluated if possible by a native language speaker but were excluded from the final database of “guideline-like” (GL) studies.

Publications identified as measuring uterine weight changes in rats or mice were reviewed, and detailed study protocol information was transcribed into an Excel spreadsheet as follows. Data entry for each study protocol was performed in a standardized format and recorded in the UTDB by PubMed Identifier, CASRN, and chemical name. Two scientists independently reviewed each manuscript for relevance and extracted information on the study protocol design and on chemical exposure effects on uterine weight. Types of information extracted from each publication and examples are provided in Table 1. Additional information about study protocols that did not fall into one of the predetermined study information categories was also recorded in corresponding “assay notes” and “response notes” columns. The lowest effect level (LEL), that is, the chemical dose that caused an active outcome (a statistically significant increase in uterine weight), was reported for any compound with a positive result. The highest dose tested (HDT) was reported for chemicals with negative results. Where possible, the LEL and HDT were recorded in units of milligrams per kilogram per day, although some studies reported alternate units such as milligrams per animal. Many publications contained multiple study protocols with different designs (e.g., comparisons of animal models, administration routes, or exposure durations). Pertinent details were recorded in the UTDB for every unique chemical/study protocol combination.

**Figure 2:**
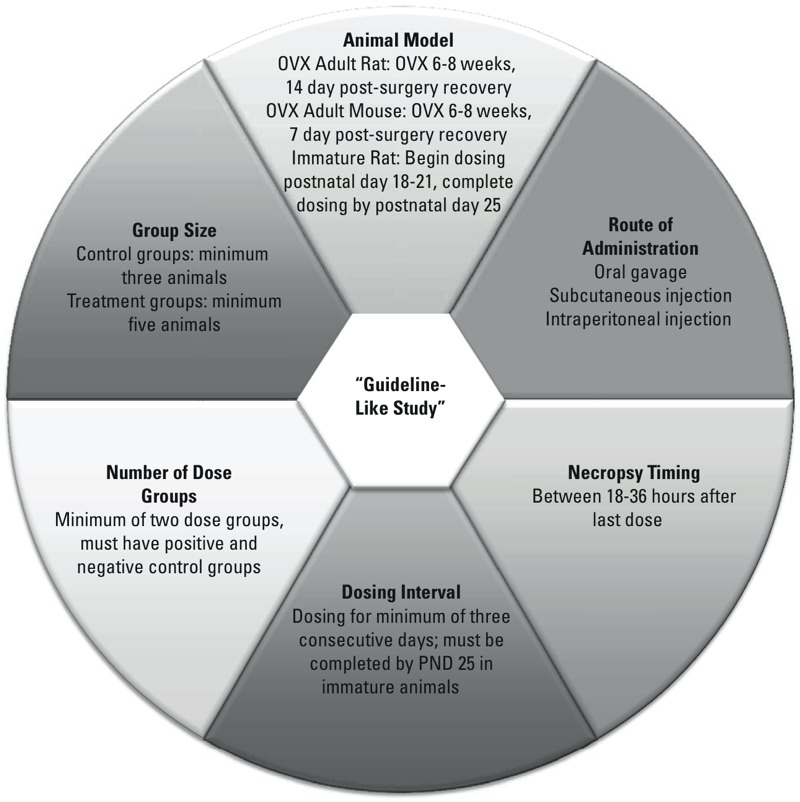
Minimum criteria for guideline-like (GL) uterotrophic studies. Abbreviations: OVX, ovariectomized; PND, postnatal day.

*Study quality evaluation.* Compliance with the uterotrophic study protocol design requirements set forth in EPA OCSPP 890.1600 (U.S. EPA 2011b) and OECD TG 440 (OECD 2004) was evaluated based on the information extracted from each publication. Two scientists independently scored each protocol for adherence to six predefined minimum criteria (MC) for a GL study. A study protocol was considered to be GL if all six of the MC shown in Figure 2 and explained in the following paragraph were met.

Acceptable animal models included immature rats, OVX adult rats, and OVX adult mice. Based on OECD recommendations, studies using immature mice were not considered to be GL because of the potential insensitivity of immature mice to weak estrogens (OECD 2004). For studies using the OVX animal model, we required the ovariectomy to have been performed between 6 and 8 weeks of age, allowing at least 14 days postsurgery before dosing for rats and 7 days postsurgery for mice to ensure adequate time for uterine tissues to regress. For immature rat studies, we required the dosing to have begun after weaning between postnatal day (PND) 18 and PND 21 and to have been completed by PND 25 (before the onset of puberty). Each positive or negative control group was required to have a minimum of three animals, and each test group was required to have a minimum of five animals. This requirement differs from those of the OECD and U.S. EPA guidelines, both of which require six animals in both control and test groups (OECD 2004; U.S. EPA 2011b). However, we found that a large number of studies that used marginally smaller group sizes fulfilled every other MC to be considered GL; therefore, we relaxed these criteria to be slightly more inclusive while ensuring sufficient statistical power. Acceptable routes of administration included oral gavage (p.o.) and subcutaneous (s.c.) and intraperitoneal (i.p.) injection, although both the OECD and U.S. EPA guidelines state that injection routes are preferred to increase the bioavailability of the test substance. We required a minimum of two dose groups treated over a minimum dosing interval of 3 consecutive days to show dose-dependent effects and establish an LEL. Finally, to ensure appropriate timing for effect evaluation, we required the necropsy to have been performed 18–36 hr after the last dose. Compared with the OECD and U.S. EPA guidelines, which specify that necropsy should occur 24 hr after the last dose (OECD 2004; U.S. EPA 2011b), this requirement was expanded to maximize the number of adherent studies. We recorded data indicating whether levels of phytoestrogen in the diet were reported, but this criterion was not incorporated into the final GL criteria because of the small number of studies reporting this information (< 5% of the 670 papers reviewed).

A score of 0 (no) or 1 (yes) was recorded for each of the minimum criteria (MC 1–6) based on whether the study protocol fulfilled that particular requirement. These scores were recorded as individual columns in the UTDB and were added to yield a total score for each study protocol. The two independent evaluations for each study protocol were compared. If the two evaluations concurred, information from that study protocol was entered into the final version of the UTDB. If the two evaluations differed, the paper was re-reviewed to identify the source of the discrepancy and reach a consensus. Only protocols that met all six criteria were considered GL. The subset of GL uterotrophic study protocols constitutes the GL uterotrophic database (GL-UTDB).

It should be noted that compliance with the MC identified above is not necessarily equivalent to a thorough assessment of overall study quality. For example, our evaluation did not consider the internal validity of each study, risk of bias, or whether the route of administration was relevant to the expected route of human exposure.

## Results

The search for uterotrophic data for the 1,812 ToxCast^TM^ compounds (http://epa.gov/comptox/toxcast/data.html) yielded > 1,000 papers, of which 670 were deemed potentially relevant based on the inclusion of uterine weight as a measured end point. From these 670 manuscripts, 2,615 individual chemical/study/protocol combinations were extracted, yielding results for 235 chemicals with unique CASRNs (http://ntp.niehs.nih.gov/pubhealth/evalatm/tox21-support/endocrine-disruptors/edhts.html). It was common for 1 paper to contain multiple study design protocols, of which only some protocols met all six MC and were included in the GL-UTDB (http://ntp.niehs.nih.gov/pubhealth/evalatm/tox21-support/endocrine-disruptors/edhts.html). The GL-UTDB contains information from 458 GL studies extracted from 93 publications, providing high-quality *in vivo* estrogenic bioactivity data for 118 chemicals with unique CASRNs (103 of which are in the ToxCast^TM^/Tox21 inventory). We included all chemicals in the studies returned by our search, some of which were not in the ToxCast^TM^ library but were included in publications that also examined ToxCast^TM^ chemicals. We performed an additional round of manual quality assurance on all study information in the GL-UTDB to confirm the accuracy of the data entry. To facilitate computational analyses, we added standardized chemical descriptor information (ChemID number, ChemID name, and molecular formula, available via http://chem.sis.nlm.nih.gov/chemidplus/) and a “protocol” variable that computationally binds multiple fields together to provide a unique identifier for each study.

*Impact of study design on uterotrophic outcome.* Six basic study designs met GL criteria depending on species (rat or mouse), route of administration (oral or injection), and use of OVX (rat or mouse) or immature (rat only) animals. The majority of studies that met GL criteria were performed using either the s.c. or the i.p. route of injection [69% (317/458)]. Both injection routes are acceptable according to OECD and U.S. EPA guidelines (OECD 2004; U.S. EPA 2011b); thus, for this analysis, “injection” refers to studies using either the s.c. or the i.p. route of administration. However, it should be noted that 99% (313/317) of the injection studies in the database used the s.c. route.

A breakdown of results by study design is provided in Table 2. Data from two chemicals commonly used as positive controls (ethinyl estradiol and estradiol) were excluded from this analysis owing to the large number of results and the inherent bias associated with their inclusion (i.e., negative results would indicate a failed “positive” control and would therefore not typically be reported), leaving 374 GL uterotrophic entries. The immature rat model was used for 76% (285/374) of the studies in the database, with 72% (204/285) of these studies using injection as the route of administration. Active outcomes were more prevalent in rat models [74% (242/327) of all rat outcomes were active] than in mouse models, in which 36% (17/47) of all outcomes were active.The OVX_mouse_oral design produced active outcomes in only 27% (6/22) of the studies. It should be noted that the selection of chemicals tested in these studies was neither random nor uniformly distributed with respect to uterotrophic bioactivity, and the performance of a particular study protocol design, particularly one with a small number of examples (e.g., OVX_rat_injection or OVX_mouse_oral), could be heavily influenced by a single publication from one laboratory testing multiple chemicals in that particular study design.

**Table 2:** Distribution of uterotrophic outcomes by study design (GL studies only).

Outcome	Imm_RatInj	Imm_RatOral	OVX_RatInj	OVX_RatOral	OVX_MouseInj	OVX_MouseOral
Number active^*a*^	147	61	29	5	11	6
Number inactive	57	20	3	5	14	16
Percent active	0.72	0.75	0.91	0.50	0.44	0.27
Percent inactive	0.28	0.25	0.09	0.50	0.56	0.73
Percent total	54.5	21.7	8.6	2.7	6.7	5.9
Abbreviations: GL, guideline-like; Imm, immature; Inj, injection (either subcutaneous or intraperitoneal); Oral, oral gavage; OVX, ovariectomized. Number active: the number of experiments reporting substances as active. Number inactive: the number of experiments reporting substances as inactive.^***a***^Data for positive controls are not included in this table.						

**Figure 3:**
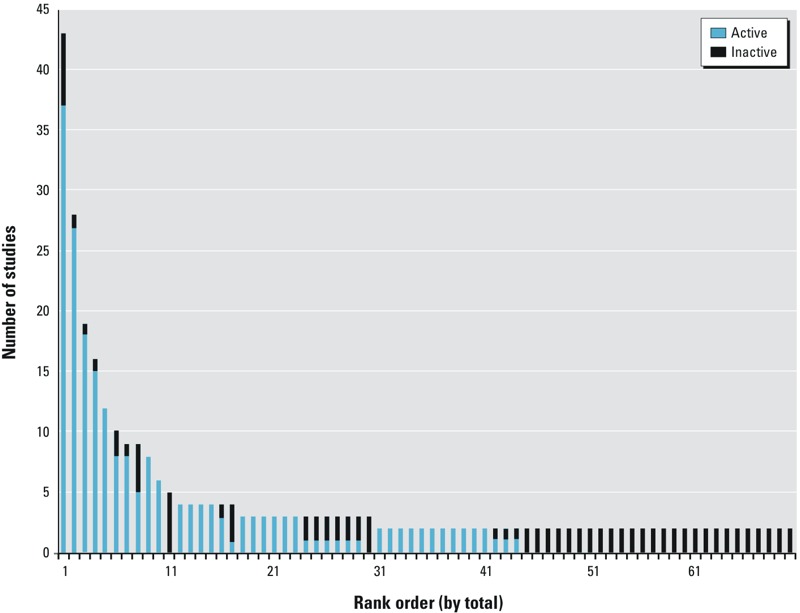
Results from uterotrophic studies for chemicals that had at least two independent guideline-like (GL) studies. Blue bars represent the number of “active” reports; black bars represent the number of “inactive” reports. Data from chemicals commonly used as positive controls (i.e., ethinyl estradiol and estradiol) were excluded from this plot.

*Reproducibility of uterotrophic outcomes.* The GL-UTDB provides an opportunity to assess both the qualitative and quantitative reproducibility of a uterotrophic assay across many chemicals tested at many different laboratories. Of the 70 chemicals in the database with at least two reported GL uterotrophic studies (Figure 3), 18 (26%) had at least one study with a discordant outcome, resulting in a chemical being classified as both “active” and “inactive” for uterotrophic bioactivity. Table 3 lists chemicals for which discordant results were reported along with the minimum reported LEL and the maximum reported HDT for each chemical. Discordant outcomes could result from differences in overall study protocol design and/or from the range of doses tested in each study. For example, the HDT from an inactive result may have been lower than the dose that would produce a tissue concentration required for bioactivity, as appears to be the case for benzophenone, permethrin, and daidzein. In other cases, the HDT for an inactive result may have been very close or equal to the minimum LEL (minLEL) for an active result, and discrepancies could be attributed to small increases that either just crossed the threshold or failed to reach statistical significance. We observed such a result for diethylstilbestrol, a known estrogenic compound, where a dose of 0.05 μg/kg/day produced a ~ 30% increase in uterine weight (*p* < 0.01) in one study (Odum et al. 2002) and produced a statistically nonsignificant increase of ~ 20% at the same dose in a different study (Tinwell and Ashby 2004), both of which used the same basic study design. However, in the same paper that reported the inactive result (Tinwell and Ashby 2004), additional experimental protocols were performed that showed significant uterotrophic activity at slightly higher diethylstilbestrol doses of 0.25 μg/kg/day. The GL-UTDB contains one additional compound, 4-nonylphenol (branched form, CASRN: 25154-52-3), that had 22 active results (minLEL of 5 mg/kg/day) and 2 inactive results [maximum HDT (maxHDT) of 80 mg/kg/day], but this compound was found to consist of a mixture of branched chains rather than to be a unique structure. Because we could not ascertain that the same form was being tested in each study, the compound was excluded from this analysis.

**Table 3:** Chemicals with discordant uterotrophic results in GL studies.

CASRN	Name	GLActive	minLEL(mg/kg/day)	GLInactive	maxHDT(mg/kg/day)
80-05-7	Bisphenol A^*a*^	37	2	6	1,000
446-72-0	Genistein^*a*^	27	1	1	5
72-43-5	Methoxychlor^*a*^	18	20	1	200
789-02-6	*o,p’*-DDT^*a*^	15	1	1	100
94-26-8	Butylparaben^*b*^	8	50	2	1,000
56-53-1	Diethylstilbestrol^*a*^	8	0.00005	1	0.00005
104-40-5	4-*n*-Nonylphenol (linear, *para*)^*a*^	5	75	4	200
140-66-9	4-*tert*-Octylphenol^*b*^	3	56	1	250
120-47-8	Ethylparaben^*a*^	1	180	3	1,000
119-61-9	Benzophenone^*a*^	1	500	2	200
99-76-3	Methylparaben^*b*^	1	55	2	800
56-55-3	Benz[*a*]anthracene^*b*^	1	1	2	300
1806-26-4	4-Octylphenol^*b*^	1	100	2	200
94-13-3	Propylparaben^*b*^	1	65	2	1,000
52645-53-1	Permethrin^*b*^	1	800	1	150
50-55-5	Reserpine^*b*^	1	3	1	3
520-36-5	Apigenin^*b*^	1	5	1	200
486-66-8	Daidzein^*b*^	1	600	1	200
Abbreviations: CASRN, Chemical Abstracts Service Registry Number; GL, guideline-like; maxHDT, maximum highest dose tested; minLEL, minimum lowest effect level; *o*,*p´*-DDT, 1-chloro-2-[2,2,2-trichloro-1-(4-chlorophenyl)ethyl]benzene.^***a***^Non-shaded chemicals had discordant results reported in assays with the same basic study design. ^***b***^Shaded chemicals had discordant uterotrophic outcomes in guideline-like study designs that differed significantly from one another.					

**Figure 4:**
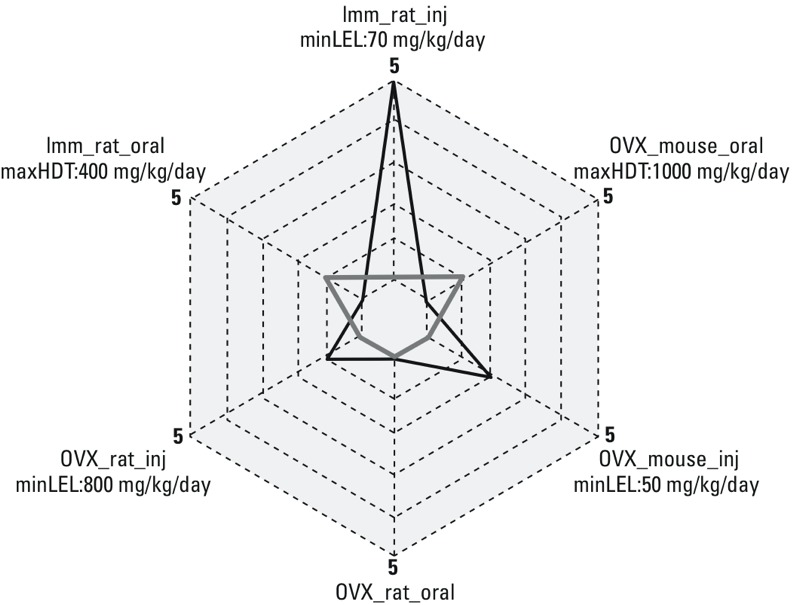
Example of butylparaben, where differences in study protocol design that may be associated with discordant uterotrophic outcomes. Numbers of active (black) and inactive (gray) outcomes are shown (dotted lines represent number of outcomes, maximum of 5 here) for butylparaben as a function of study design. The minimum lowest effect level (minLEL) is reported for the 8 active outcomes (5 Imm_rat_inj, 2 OVX_mouse_inj, 1 OVX_rat_inj) and the maximum highest dose tested (maxHDT) is reported for the 2 inactive outcomes (1 Imm_rat_oral, 1 OVX_mouse_oral). Abbreviations: Imm, immature; inj, injection (either subcutaneous or intraperitoneal); oral, oral gavage; OVX, ovariectomized.

Of the 18 chemicals listed in Table 3, 10 (56%, shaded rows in the table) had discordant uterotrophic outcomes that may be attributable to differences in study protocol design. The results obtained from testing butylparaben provide an example of how study design can affect uterotrophic outcomes, as shown in the radar plot in Figure 4. In the case of this compound, all eight active results were reported in the three study protocol designs using s.c. injection as the route of administration (immature rat, OVX rat, OVX mouse), whereas inactive results were reported for both study protocol designs that used oral dosing (immature rat, OVX mouse). In all three injection protocols, the minLEL reported was well below the maximum highest dose tested in the oral dosing protocols. Similar radar plots for each chemical in Table 3, illustrating the relationship between study protocol design and outcome, are provided in Supplemental Material, Figure S1.

**Figure 5:**
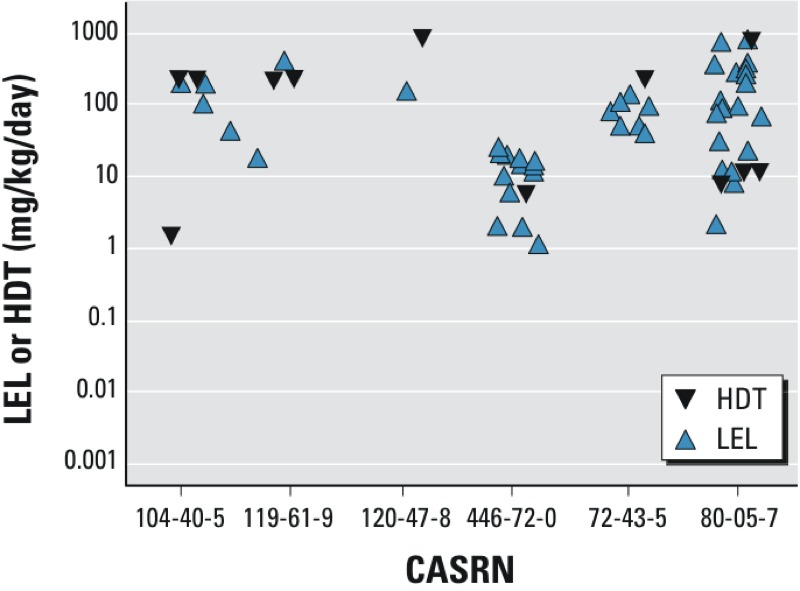
LELs and HDTs for six chemicals with discordant results in the Immature_Rat_Injection study design. Markers reflect lowest effect levels (LELs) for chemicals classified as “active” in the uterotrophic bioassay (blue markers), and highest dose tested (HDT) for those with “inactive” uterotrophic outcomes (black markers). CASRNs: 104-40-5, 4-*n*-nonylphenol (linear, *para*); 119-61-9, benzophenone; 120-47-8, ethylparaben; 446-72-0, genistein; 72-43-5, methoxychlor; 80-05-7, bisphenol A.

Discordant outcomes were reported for the eight chemicals in the nonshaded rows in Table 3 in studies that were performed using the same basic study design. Uterotrophic outcomes were compared to determine whether the HDT for inactive outcomes was below the LEL reported for active outcomes, in which case the results would actually support one another. For chemicals that had discordant outcomes reported for studies performed using the same study design, it was common for the HDT to be above LEL doses reported in other studies, although the differences between these values were typically less than one order of magnitude. Most studies in the UTDB and the GL-UTDB typically used no more than four log-spaced doses, resulting in poor resolution of LELs (generally defined as > 20% increase in wet uterine weight, *p* < 0.05), which could explain LELs and HDTs reported at similar doses. However, reports of inactive results obtained at doses well above all reported LELs are difficult to reconcile. Figure 5 shows discordant results for chemicals tested using the same basic study design: immature rat and s.c. injection, which was the most common design and correspondingly had the highest number of discrepancies. Bisphenol A (BPA, CASRN 80-05-7) provides a good example of the high degree of variability that can be seen in the uterotrophic bioassay, with BPA classified as “active” in one study using the immature rat model when administered by s.c. injection at 2 mg/kg/day (Takeyoshi 2006), and “inactive” in another study using the same model when adminstered by s.c. injection at 1,000 mg/kg/day (An et al. 2002).

*Chemicals with independently reproducible uterotrophic outcomes.* Thirty-six chemicals (24 active, 12 inactive) showed reproducible results in two or more independent GL uterotrophic studies (Table 4). The minLEL and maxHDT are reported in milligrams per kilogram per day; however, this information cannot necessarily be translated into expected potency values because it is inherently limited for some compounds by the dose ranges selected in the studies. Furthermore, there are studies with potentially lower LELs than those reported in Table 4 that were reported in terms of milligrams per animal per day or in terms of total dose. For consistency, we used the minLEL from studies that reported units of milligrams per kilogram per day unless the only studies reporting outcomes for a given chemical reported doses in units other than milligrams per kilogram per day.

**Table 4:** Chemicals with independently reproduced concordant guideline-like uterotrophic results.

CASRN	Name	GL Active	GLInactive	Bioactivity	minLEL (mg/kg/day)	maxHDT (mg/kg/day)
50-28-2	Estradiol	25	0	Active	0.00001	NA
57-63-6	Ethinyl Estradiol	59	0	Active	0.0001	NA
72-33-3	Mestranol	3	0	Active	0.00008^*a*^	NA
50-27-1	Estriol	4	0	Active	0.002^*a*^	NA
10540-29-1	Tamoxifen	12	0	Active	0.01	NA
57-91-0	Alfatradiol	2	0	Active	0.4	NA
68-22-4	Norethindrone	2	0	Active	2	NA
53-16-7	Estrone	9	0	Active	2	NA
474-86-2	Equilin	2	0	Active	2	NA
17924-92-4	Zearalenone	4	0	Active	2	NA
50-41-9	Clomiphene citrate	2	0	Active	2	NA
1478-61-1	Bisphenol AF	4	0	Active	4	NA
58-18-4	Methyltestosterone	3	0	Active	10	NA
80-09-1	Bisphenol S	2	0	Active	20	NA
77-40-7	Bisphenol B	2	0	Active	20	NA
599-64-4	4-Cumylphenol	2	0	Active	20	NA
521-18-6	Dihydrotestosterone	3	0	Active	20	NA
104-43-8	4-Dodecylphenol	3	0	Active	40	NA
98-54-4	4-*tert*-Butylphenol	2	0	Active	100	NA
131-56-6	2,4-Dihydroxybenzophenone	3	0	Active	100	NA
80-46-6	4-*tert*-Amylphenol	4	0	Active	200	NA
5153-25-3	2-Ethylhexyl 4-hydroxybenzoate	2	0	Active	200	NA
131-55-5	Benzophenone-2	6	0	Active	200	NA
556-67-2	Octamethylcyclotetrasiloxane	3	0	Active	250	NA
51630-58-1	Fenvalerate	0	2	Inactive	NA	80
1461-22-9	Tributyltin chloride	0	2	Inactive	NA	200
99-96-7	4-Hydroxybenzoic acid	0	2	Inactive	NA	1,000
87-86-5	Pentachlorophenol	0	2	Inactive	NA	1,000
84-75-3	Dihexyl phthalate	0	2	Inactive	NA	1,000
84-74-2	Dibutyl phthalate	0	2	Inactive	NA	1,000
84-61-7	Dicyclohexyl phthalate	0	2	Inactive	NA	1,000
61-82-5	Amitrole	0	2	Inactive	NA	1,000
520-18-3	Kaempferol	0	3	Inactive	NA	1,000
117-81-7	Bis(2-ethylhexyl) phthalate	0	2	Inactive	NA	1,000
103-23-1	Bis(2-ethylhexyl) hexanedioate	0	2	Inactive	NA	1,000
84-66-2	Diethyl phthalate	0	2	Inactive	NA	2,000
Abbreviations: CASRN, Chemical Abstracts Service Registry Number; GL, guideline-like; maxHDT, maximum highest dose tested; minLEL, minimum lowest effect level; NA, not applicable.^***a***^The minLEL (for active chemicals) and maxHDT (for inactive chemicals) are shown in units of mg/kg/day, except in the cases of mestranol and estriol, where the only reported minLELs were in mg/rat/day.						

The active compounds included steroid pharmaceuticals commonly used as positive controls and multiple BPA analogues, and the inactive compounds included several phthalates. In addition, tamoxifen and clomiphene citrate (Mirkin and Pickar 2015), 2 well-known selective estrogen receptor modulators with both agonist and antagonist activities were included in the actives list. There were 2 additional active compounds (gibberellic acid and tiratricol) with LELs in more than one protocol, but they were part of the same study by the same laboratory and were therefore not considered to be independently reproduced. Similarly, 13 inactive compounds were negative in multiple protocols run as part of one study and are therefore not shown in Table 4. Ten of these 13 were from a study that was part of an OECD validation that examined both s.c. and p.o. routes of administration in immature rats (Ohta et al. 2012).

## Discussion

U.S. and international regulations require the testing of chemicals to detect potential endocrine disruptors, but there are thousands of chemicals in commerce for which no data are currently available. *In vitro* HTS screening assays have been developed to fill some of these data gaps in a timely and cost-effective manner, but in order to use these data for hazard identification purposes, the usefulness and limitations of these *in vitro* assays must be carefully evaluated. To better understand and characterize the relationship between the *in vitro* and *in vivo* activity of potential endocrine disruptors, we developed a curated database of high-quality *in vivo* data relevant to estrogen receptor agonism from the available literature. We focused specifically on the estrogen receptor pathway because of the large number of chemicals that have been tested in the uterotrophic assay, an *in vivo* screening test that has undergone international validation by OECD (Kanno et al. 2001, 2003; Owens and Koëter 2003) and is included in the U.S. EPA’s EDSP Tier 1 battery (U.S. EPA 2012).

Our database and the accompanying analyses and chemical lists represent the first of at least three such efforts to describe the *in vivo* endocrine activity of chemicals encompassing the estrogen, androgen, and thyroid pathways. This curated information serves as a valuable anchoring point for assessing the impact of study design on test results, the reproducibility of chemical activity, and the performance of *in vitro*/computational approaches. We have provided herein a transparent outline of the strategies used to identify rodent uterotrophic studies. Data were extracted from the literature, reviewed by two independent reviewers, and assigned a score based on minimum criteria derived to mimic the study parameters defined in U.S. EPA and OECD test guidelines accepted by U.S. and international regulatory authorities. In total, > 40 parameters were extracted from each study to allow downstream analyses of their relative impact on study results. The large number of chemicals included in the GL-UTDB far exceeds the total of seven chemicals examined in the OECD validation of the uterotrophic assay (OECD 2007) and may provide a more robust assessment of the experimental variability associated with this *in vivo* test method.

Our results revealed substantive variability in the *in vivo* outcomes for chemicals tested more than once, which will be valuable information for characterizing the relevance and reliability of proposed alternatives. We analyzed sources of variability in outcomes and study designs and found that the observed discordances were largely attributable to differences in study design, which were most often based on differences in dosing route or maximum dose tested. The substantially higher number of positive outcomes in injection studies than in oral studies highlights the need to understand the impact of exposure route and metabolism on actual tissue dose as well as the need to employ reverse dosimetry to more accurately extrapolate from *in vitro* to *in vivo* bioactivity (Chang et al. 2014; Wetmore 2015; Wetmore et al. 2012). When establishing performance metrics for any alternative test method, it is important to consider both the inherent variability of the *in vivo* method and the variability associated with using different protocols. Examples of inherent variability include potential false negatives in the uterotrophic assay because of the limited number of animals used in each group or the relatively short duration of a study, and the variability in control uterus weights (Ashby and Odum 2004; Christian et al. 1998). An alternative method, such as the ToxCast^TM^ assays, may realistically be expected to predict the true response but not necessarily the associated *in vivo* experimental variability (Browne et al. 2015).

We have focused on high-quality studies that met all of our minimum criteria to be considered GL. However, we have included all the necessary information for others to reanalyze the data in a more inclusive or more stringent fashion as fits their needs, whether those needs are research- or regulatory-related. There are undoubtedly a number of reliable studies in the UTDB that did not meet all six of the minimum criteria whose data could be included in future analyses; these studies include positive results from assays performed in immature mice (Ding et al. 2010; Hossaini et al. 2000; Tinwell et al. 2000) or single-dose studies that were part of the OECD validation (Kim et al. 2005).

## Conclusion

We anticipate that the uterotrophic results compiled for this manuscript will serve as a valuable resource for understanding sources of *in vivo* study variability and reproducibility, for providing biological context for data generated from *in vitro* estrogen receptor agonist assays, and for anchoring predictive *in silico* models for estrogenic bioactivity via identification of estrogen agonist reference chemicals.

## Supplemental Material

(193 KB) PDF
