# Supplementary material for: A Curated Database of Rodent Uterotrophic Bioactivity
Source: Environ Health Perspect. 2015 Oct 2;124(5):556–62. doi: 10.1289/ehp.1510183 (PMC4858395; doi:10.1289/ehp.1510183)
Supplement: (193 KB) PDF [file ehp.1510183.s001.acco.pdf]

Environ Health Perspect
DOI: 10.1289/ehp.1510183

Note to Readers: *EHP* strives to ensure that all journal content is accessible to all readers. However, some figures and Supplemental Material published in *EHP* articles may not conform to 508 standards due to the complexity of the information being presented. If you need assistance accessing journal content, please contact ehp508@niehs.nih.gov. Our staff will work with you to assess and meet your accessibility needs within 3 working days.

Supplemental Material

A Curated Database of Rodent Uterotrophic Bioactivity

Nicole C. Kleinstreuer, Patricia C. Ceger, David G. Allen, Judy Strickland, Xiaoqing Chang,

Jonathan T. Hamm, and Warren M. Casey

Table of Contents

Supplemental Figure S1: Radar Plots for Chemical with Discordant Uterotrophic Study Designs. Numbers of active (black) and inactive (red) outcomes are shown as a function of study design (corresponding to Table 3). The maximum number of studies differs for each chemical and is shown in blue. The minimum lowest effect level (minLEL) is reported for active outcomes and the maximum highest dose tested (maxHDT) is reported for inactive outcomes. Imm - immature, OVX -ovariectomized, Inj -injection (either subcutaneous or intraperitoneal), Oral – oral gavage.

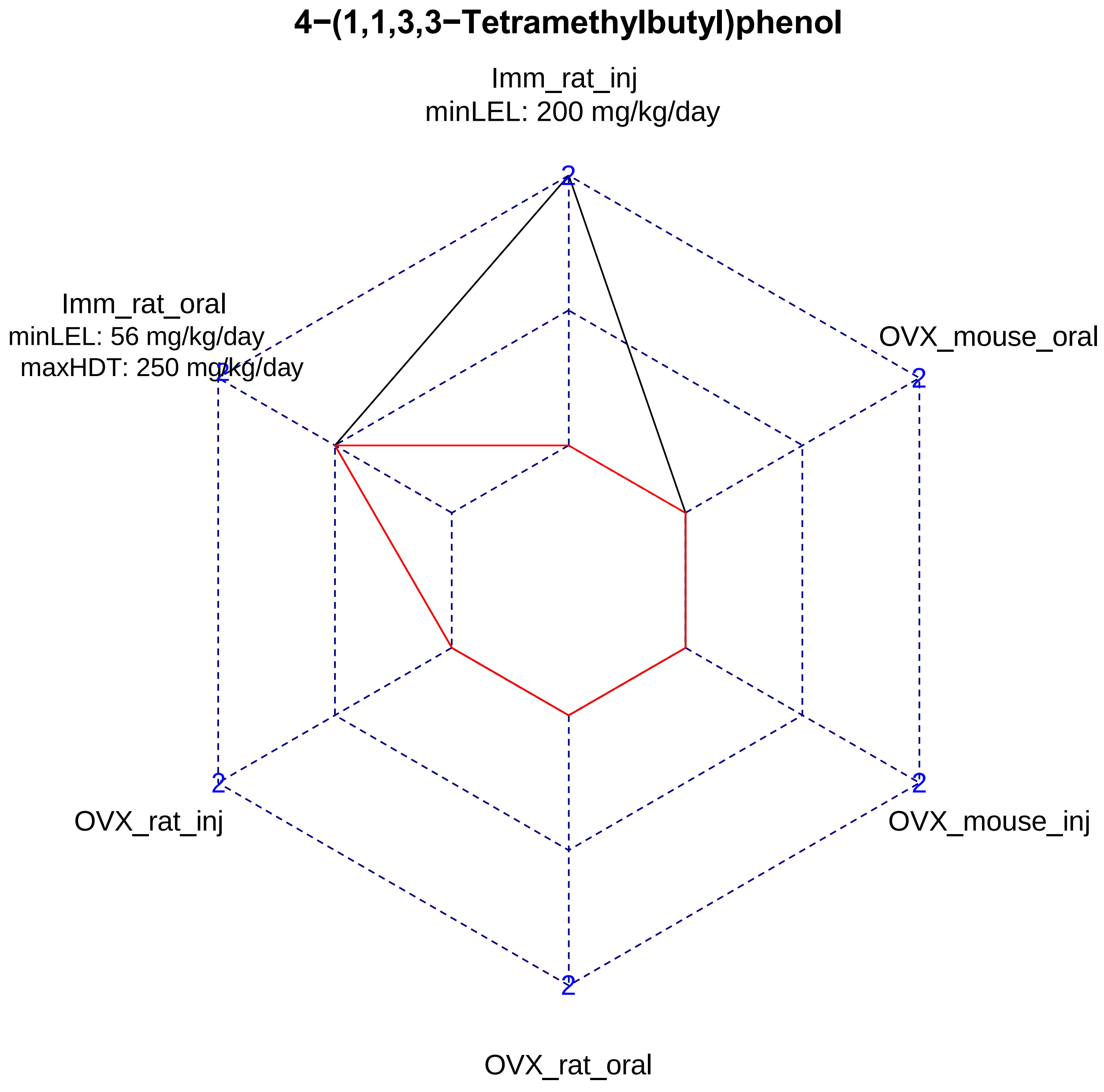
4−(1,1,3,3−Tetramethylbutyl)phenol
Imm_rat_inj
minLEL: 200 mg/kg/day
Imm_rat_oral
minLEL: 56 mg/kg/day
maxHDT: 250 mg/kg/day
OVX_mouse_oral
OVX_rat_inj
OVX_mouse_inj
OVX_rat_oral
2
2
2
2
2
2

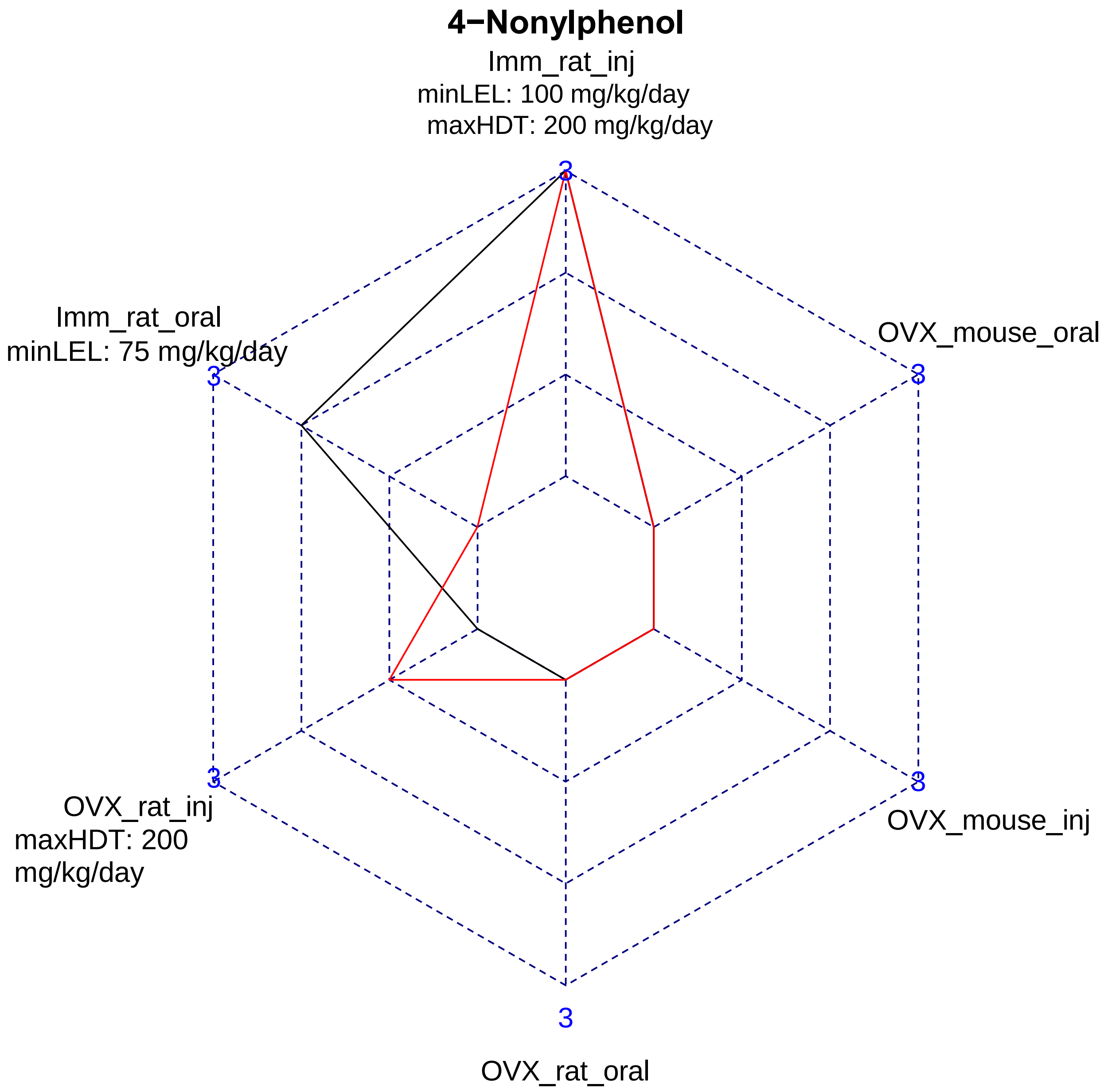
4−Nonylphenol
Imm_rat_inj
minLEL: 100 mg/kg/day
maxHDT: 200 mg/kg/day
OVX_mouse_oral
OVX_mouse_inj
OVX_rat_oral
OVX_rat_inj
maxHDT: 200
mg/kg/day
Imm_rat_oral
minLEL: 75 mg/kg/day
3
3
3
3
3
3

4−Octylphenol

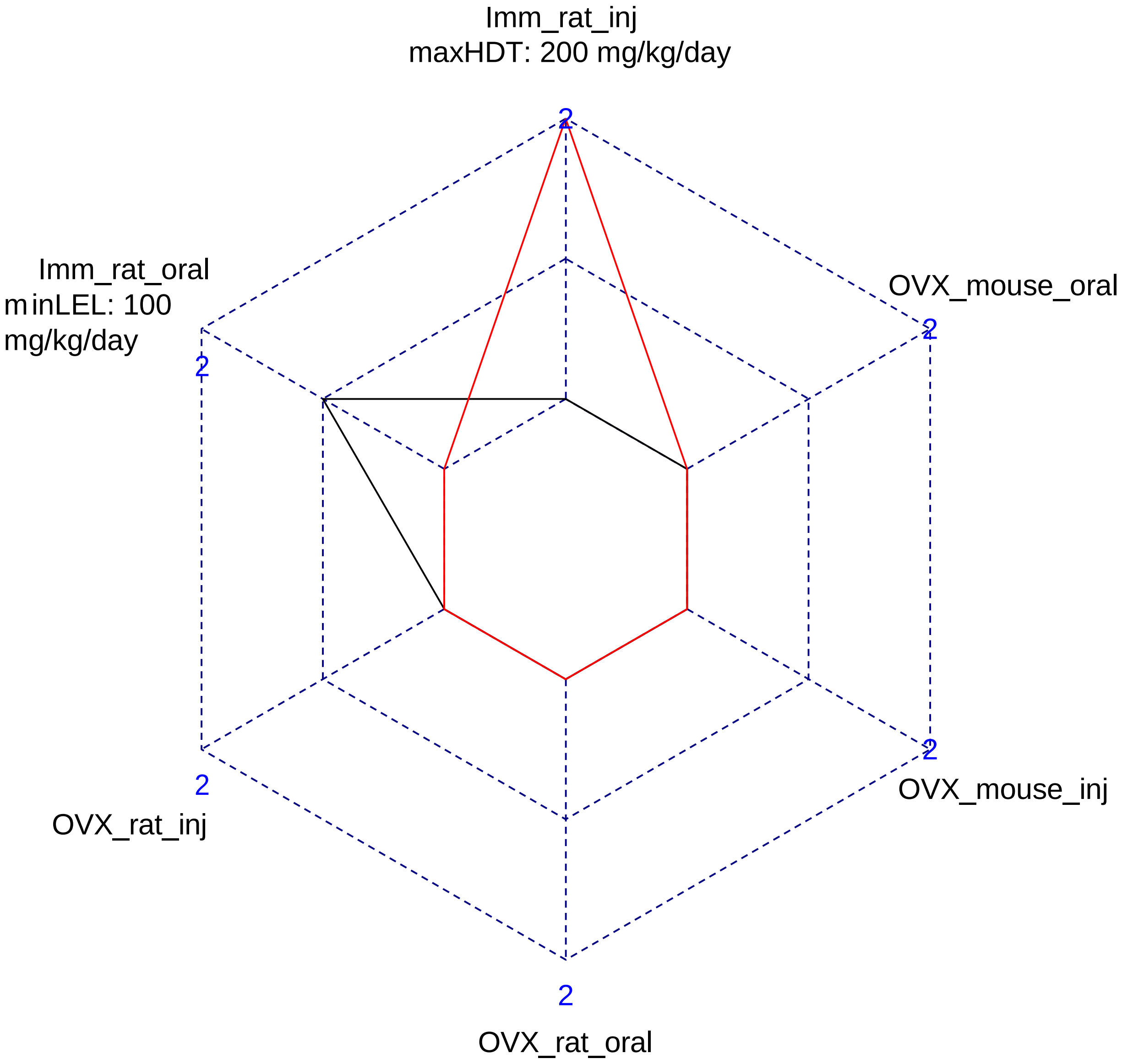

Apigenin

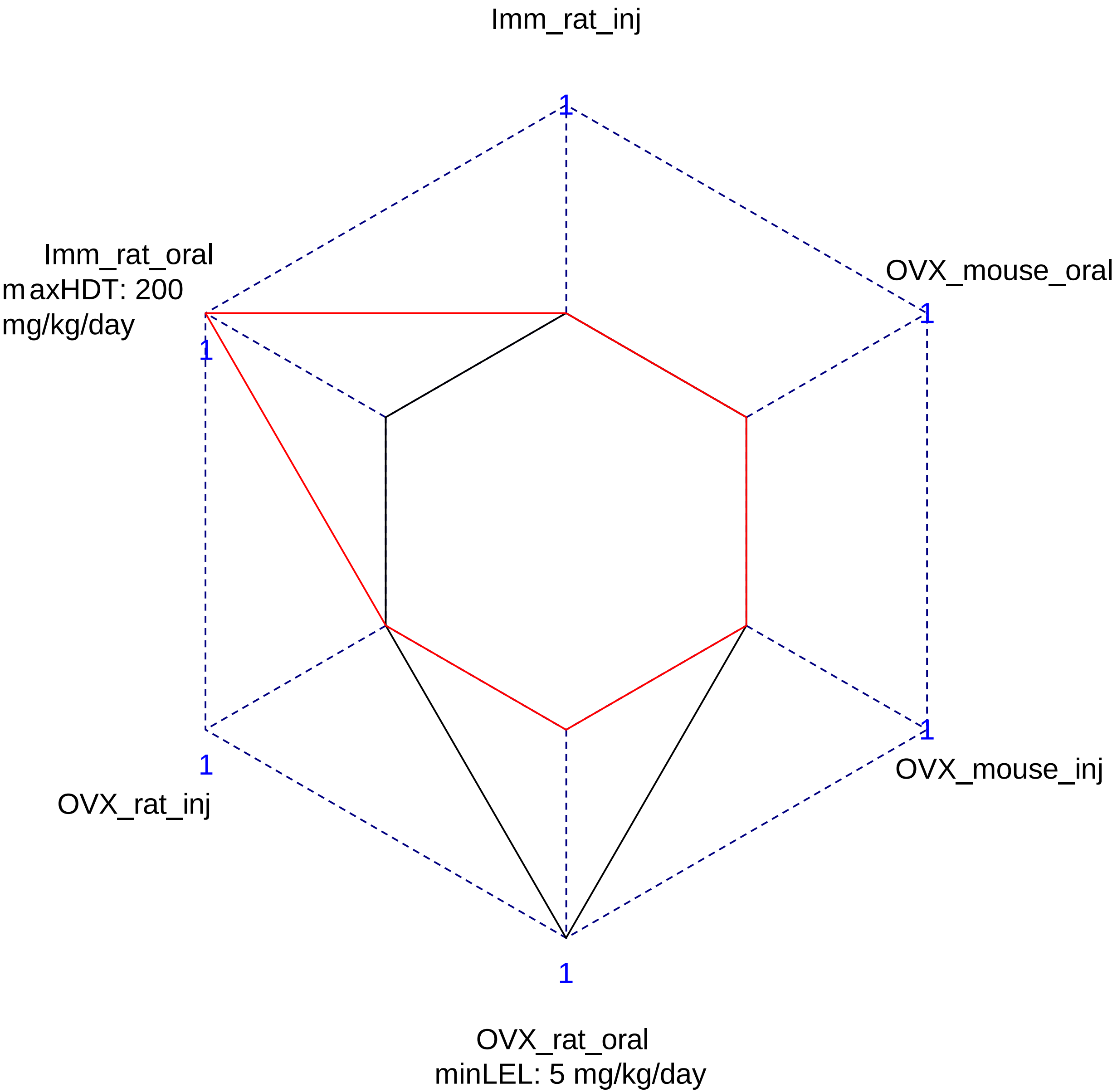

Imm_rat_inj
1
OVX_mouse_oral
1
OVX_mouse_inj
1
OVX_rat_oral
minLEL: 5 mg/kg/day
1
OVX_rat_inj
1
Imm_rat_oral
maxHDT: 200
mg/kg/day
1

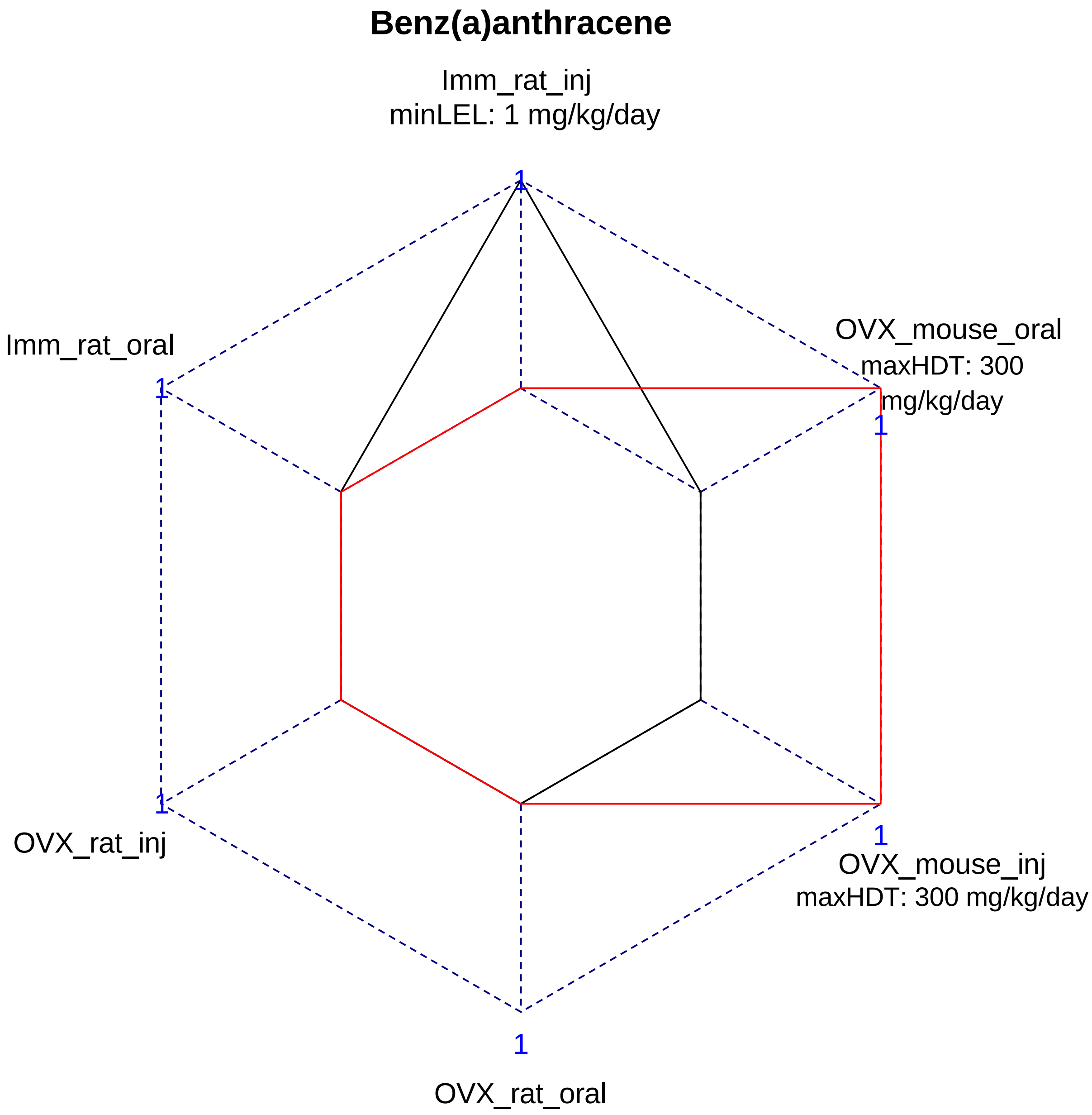
Benz(a)anthracene
Imm_rat_inj
minLEL: 1 mg/kg/day
1
OVX_mouse_oral
maxHDT: 300
mg/kg/day
1
Imm_rat_oral
1
OVX_rat_inj
1
1
OVX_mouse_inj
maxHDT: 300 mg/kg/day
1
OVX_rat_oral

Benzophenone

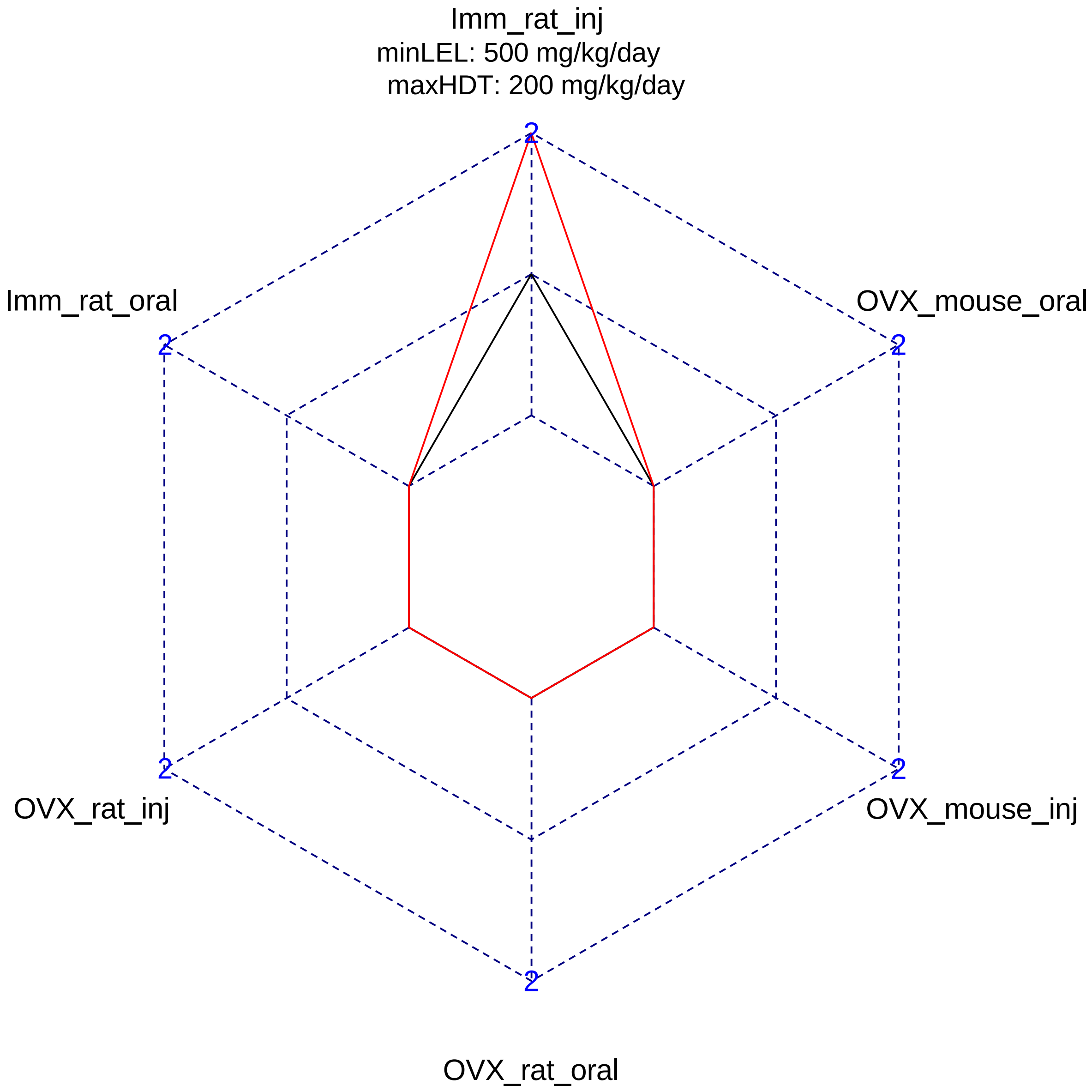

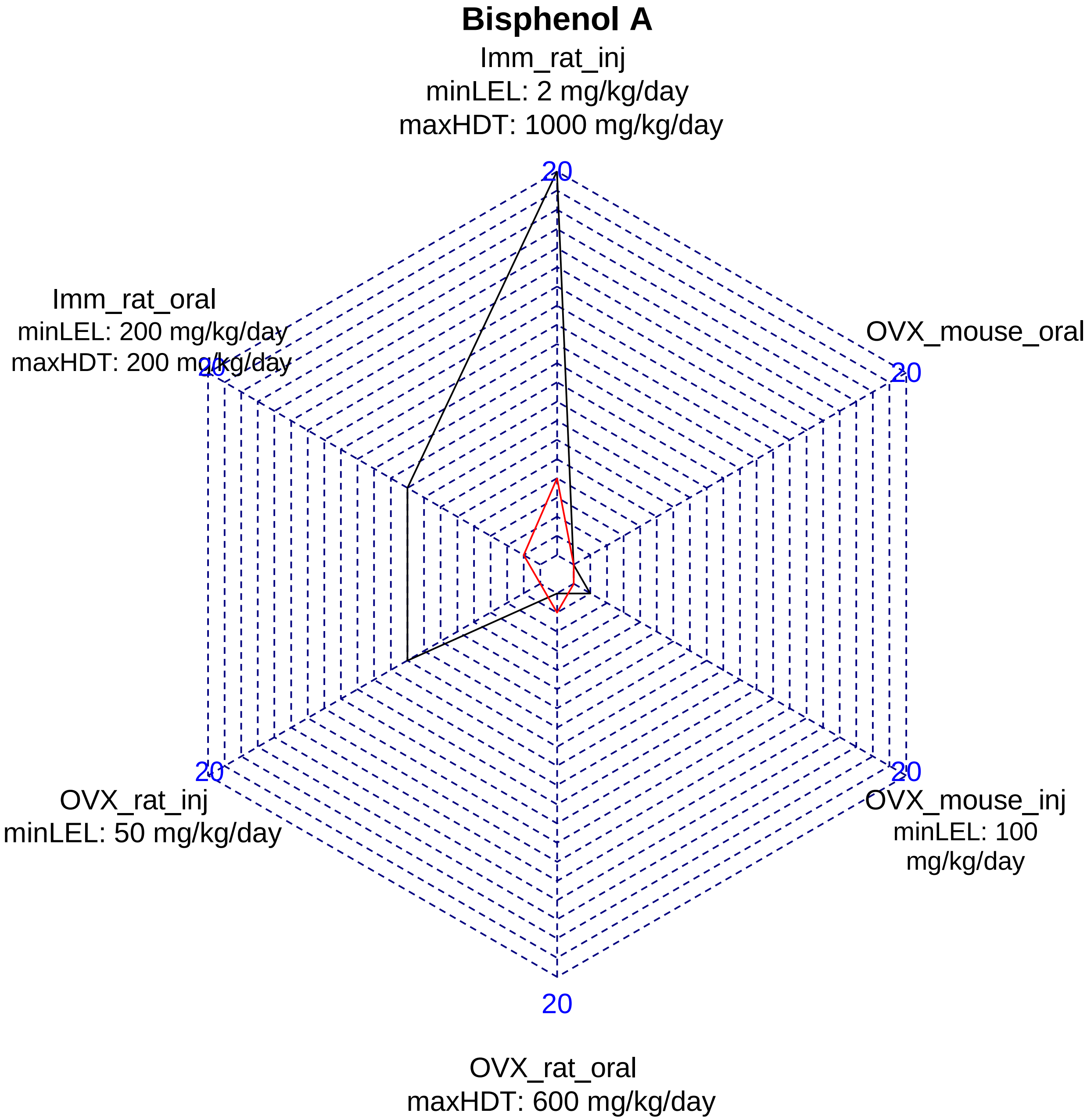

Bisphenol A
Imm_rat_inj
minLEL: 2 mg/kg/day
maxHDT: 1000 mg/kg/day
20
OVX_mouse_oral
20
OVX_mouse_inj
minLEL: 100 mg/kg/day
20
OVX_rat_oral
maxHDT: 600 mg/kg/day
20
OVX_rat_inj
minLEL: 50 mg/kg/day
20
Imm_rat_oral
minLEL: 200 mg/kg/day
maxHDT: 200 mg/kg/day
20

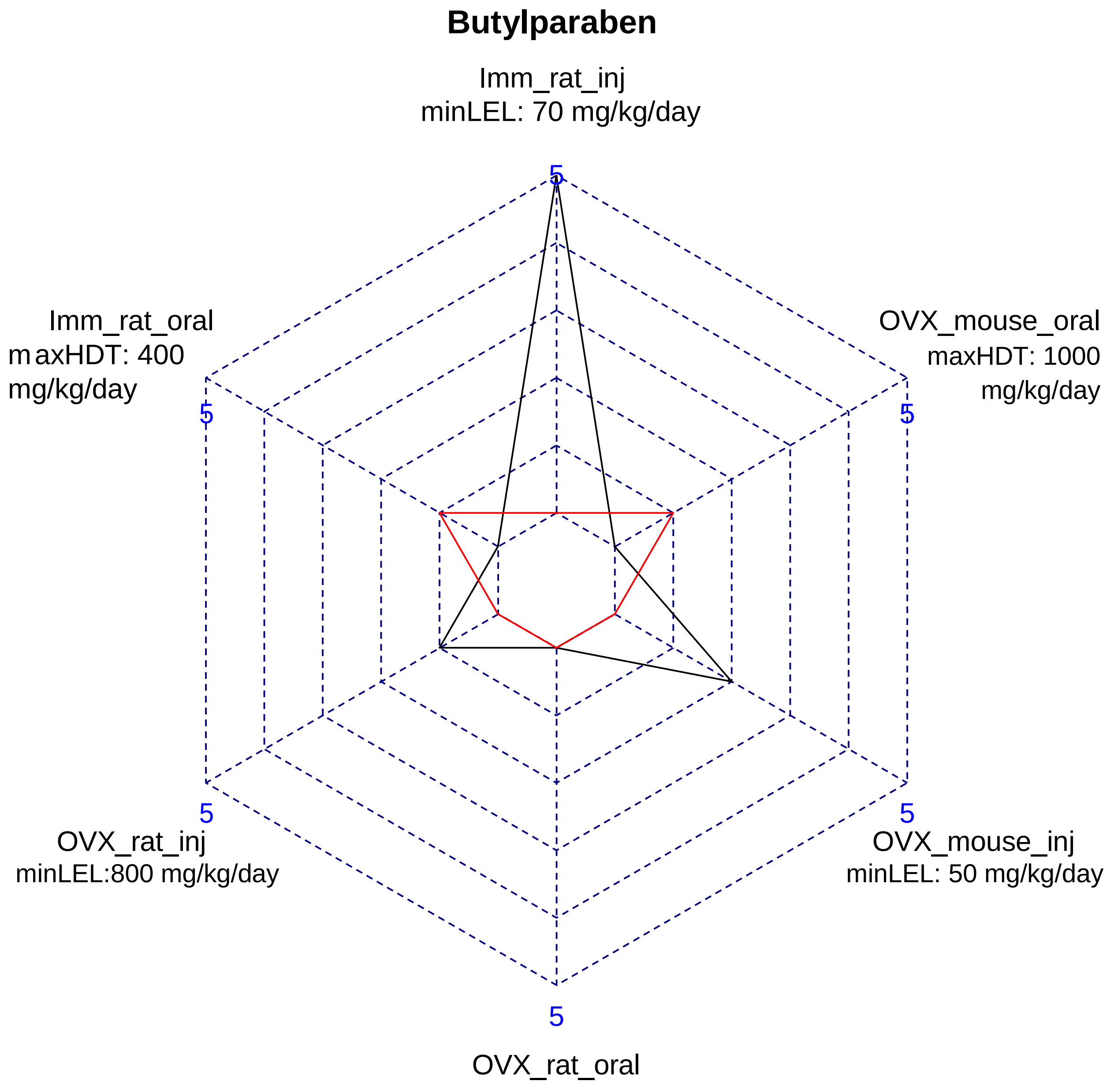

Butylparaben
Imm_rat_inj
minLEL: 70 mg/kg/day
OVX_mouse_oral
maxHDT: 1000
mg/kg/day
OVX_mouse_inj
minLEL: 50 mg/kg/day
OVX_rat_oral
OVX_rat_inj
minLEL:800 mg/kg/day
Imm_rat_oral
m axHDT: 400
mg/kg/day
5
5
5
5
5
5

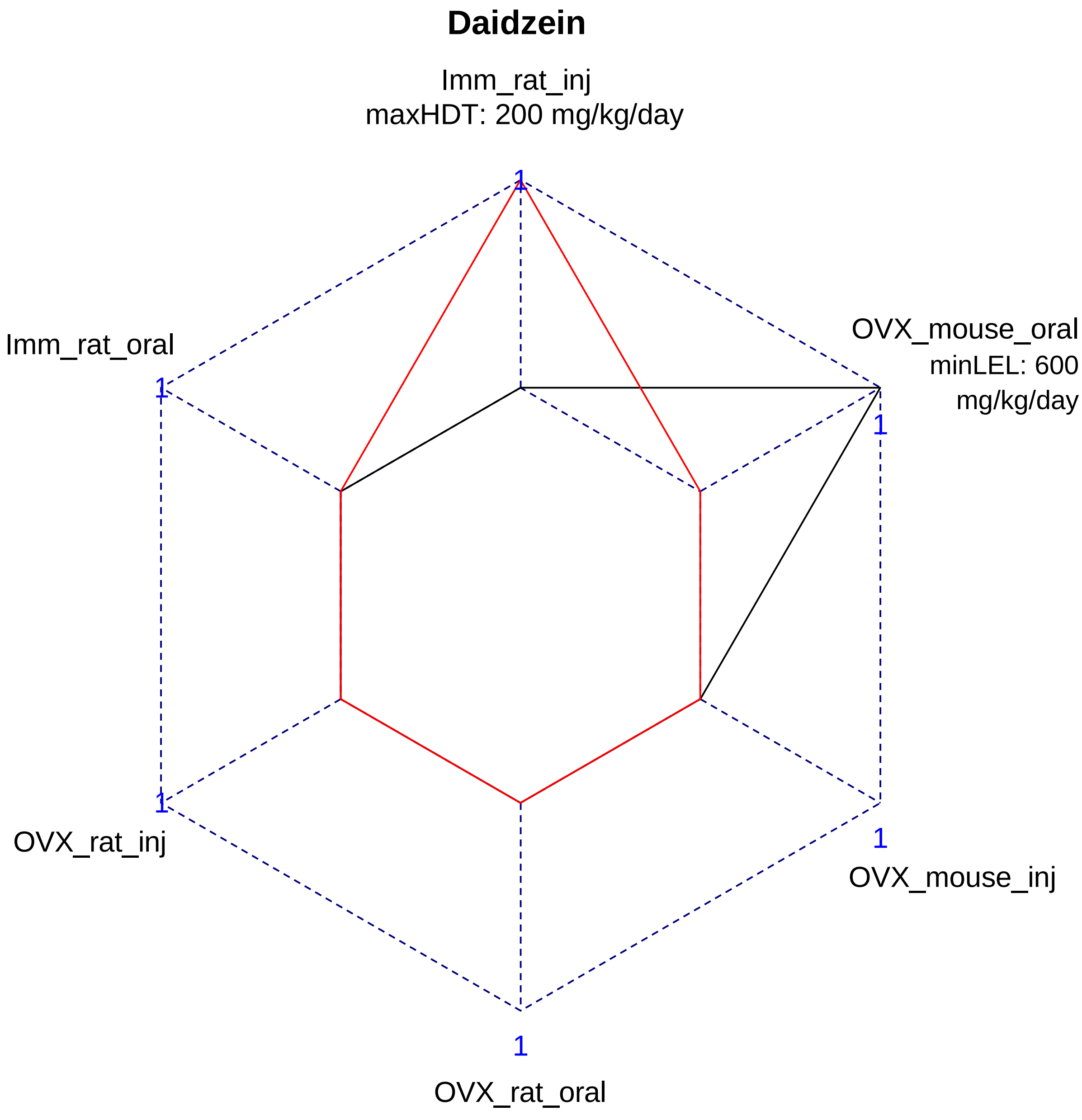
Daidzein
Imm_rat_inj
maxHDT: 200 mg/kg/day
OVX_mouse_oral
minLEL: 600
mg/kg/day
Imm_rat_oral
OVX_rat_inj
OVX_mouse_inj
OVX_rat_oral
1
1
1
1
1
1

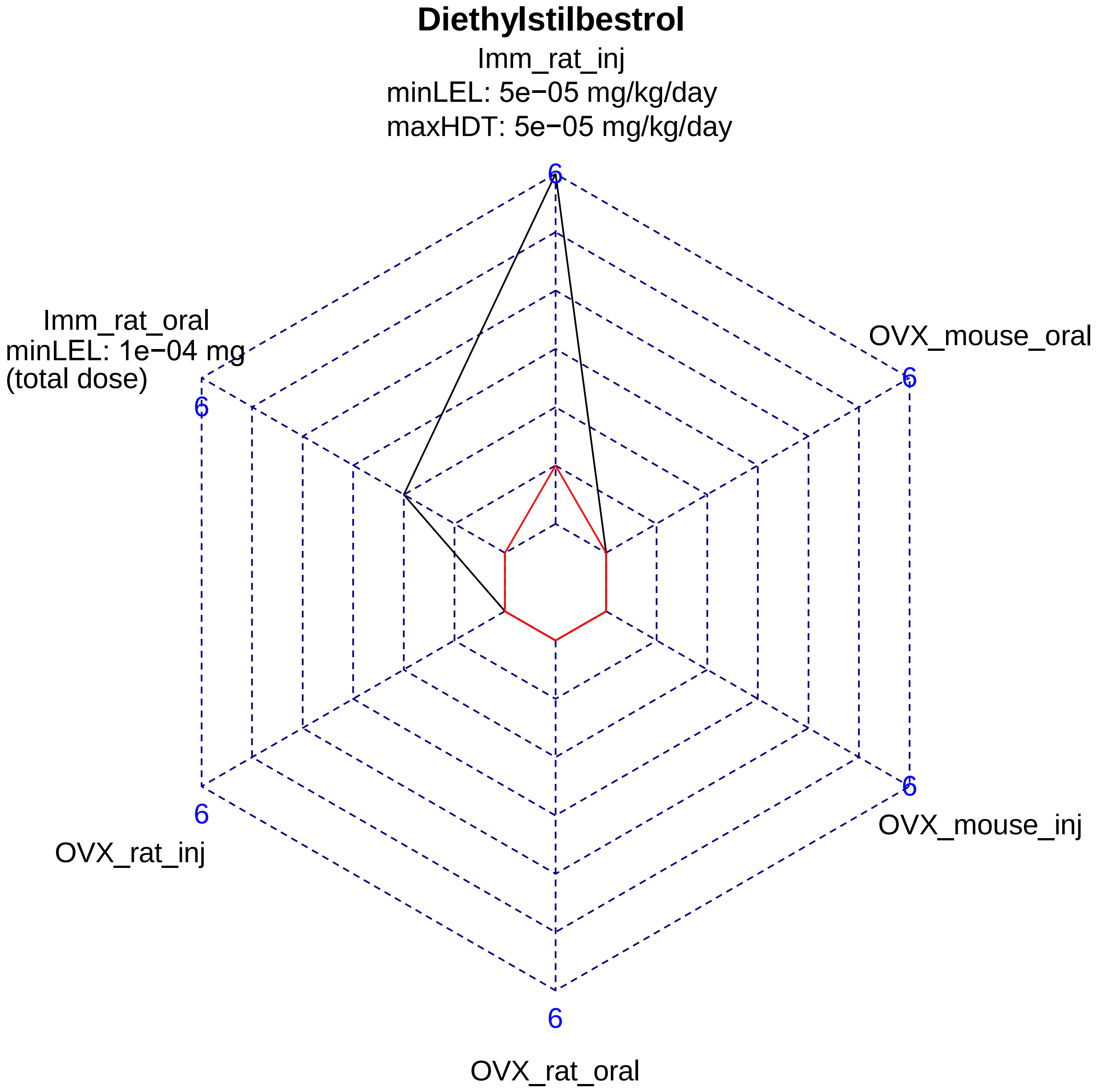
Diethylstilbestrol
Imm_rat_inj
minLEL: 5e−05 mg/kg/day
maxHDT: 5e−05 mg/kg/day
OVX_mouse_oral
Imm_rat_oral
minLEL: 1e−04 mg
(total dose)
6
6
6
6
6
6
OVX_mouse_inj
OVX_rat_inj
OVX_rat_oral

Ethylparaben

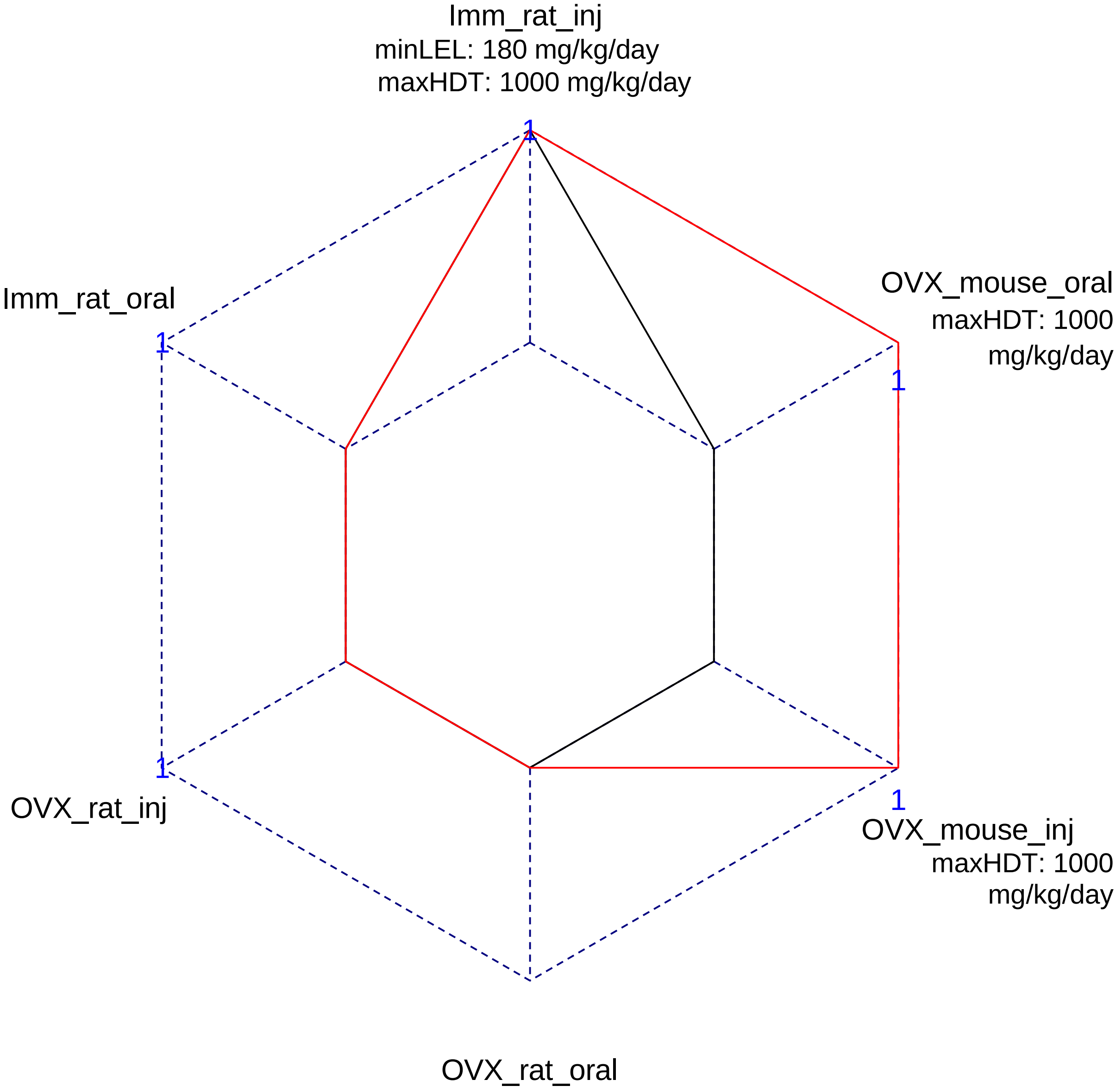

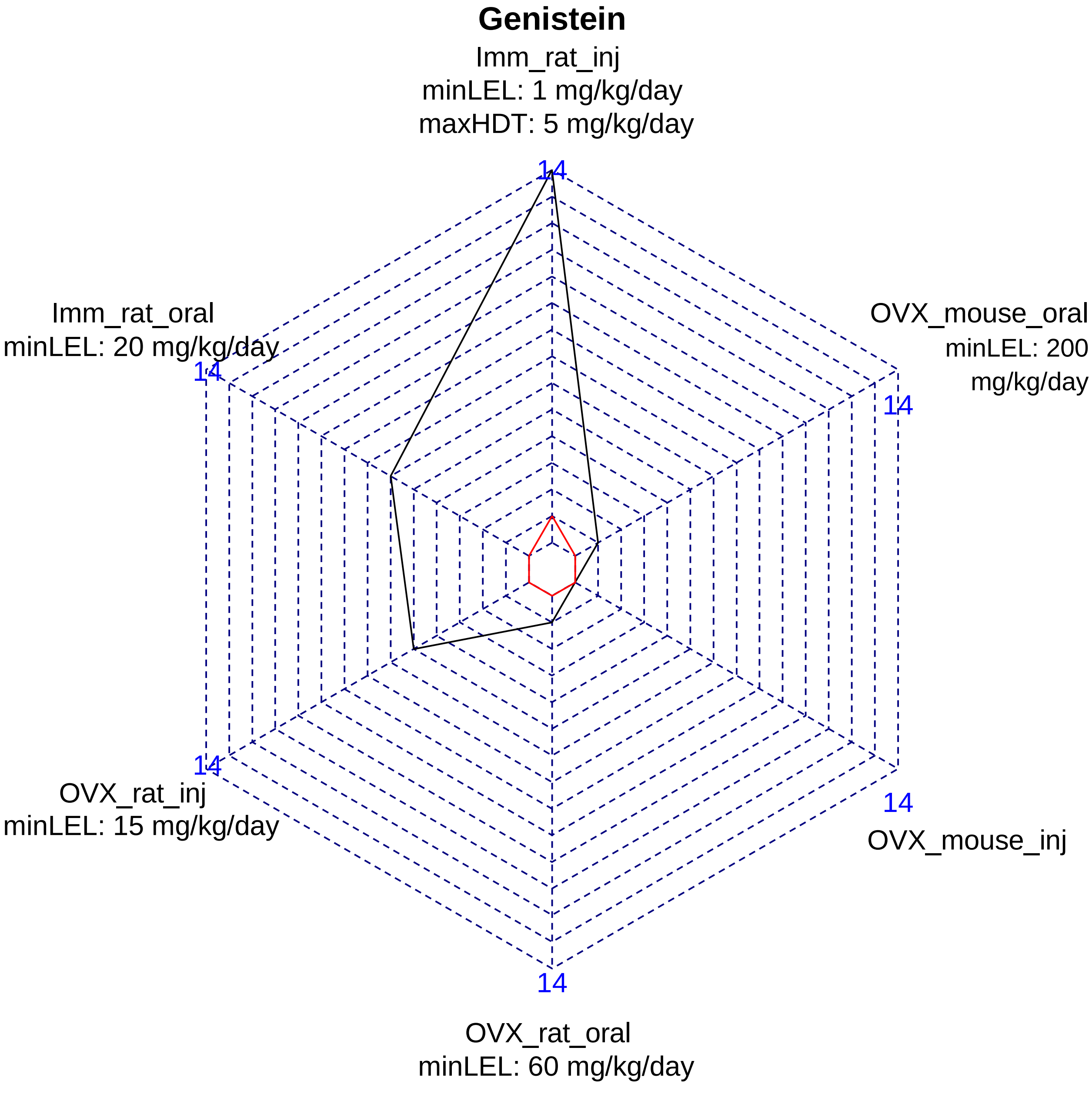
Genistein
Imm_rat_inj
minLEL: 1 mg/kg/day
maxHDT: 5 mg/kg/day
14
OVX_mouse_oral
minLEL: 200
mg/kg/day
14
14
OVX_mouse_inj
14
OVX_rat_oral
minLEL: 60 mg/kg/day
14
OVX_rat_inj
minLEL: 15 mg/kg/day
14
Imm_rat_oral
minLEL: 20 mg/kg/day

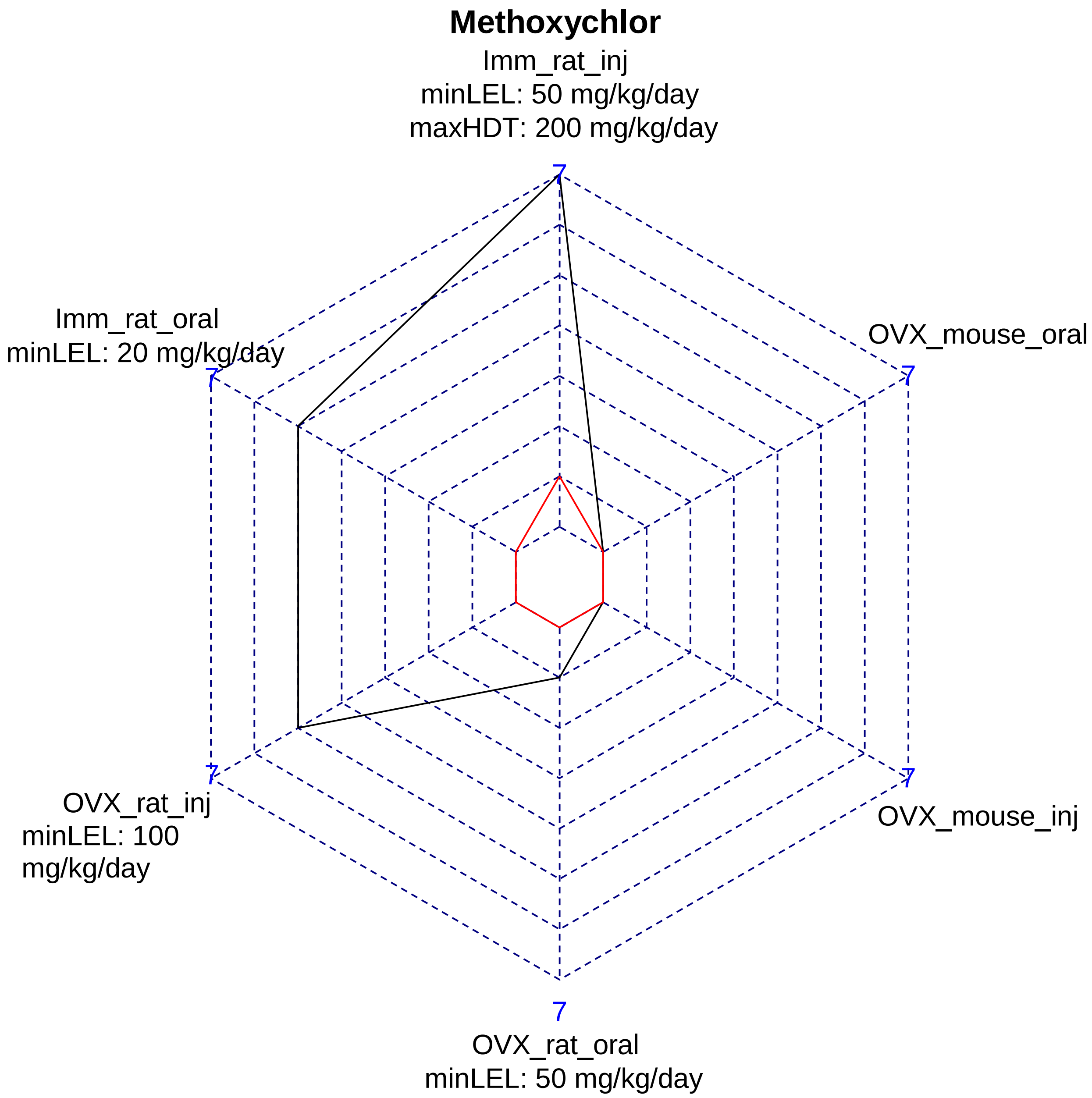
Methoxychlor
Imm_rat_inj
minLEL: 50 mg/kg/day
maxHDT: 200 mg/kg/day
7
OVX_mouse_oral
7
Imm_rat_oral
minLEL: 20 mg/kg/day
7
7
OVX_mouse_inj
7
OVX_rat_inj
minLEL: 100
mg/kg/day
7
OVX_rat_oral
minLEL: 50 mg/kg/day

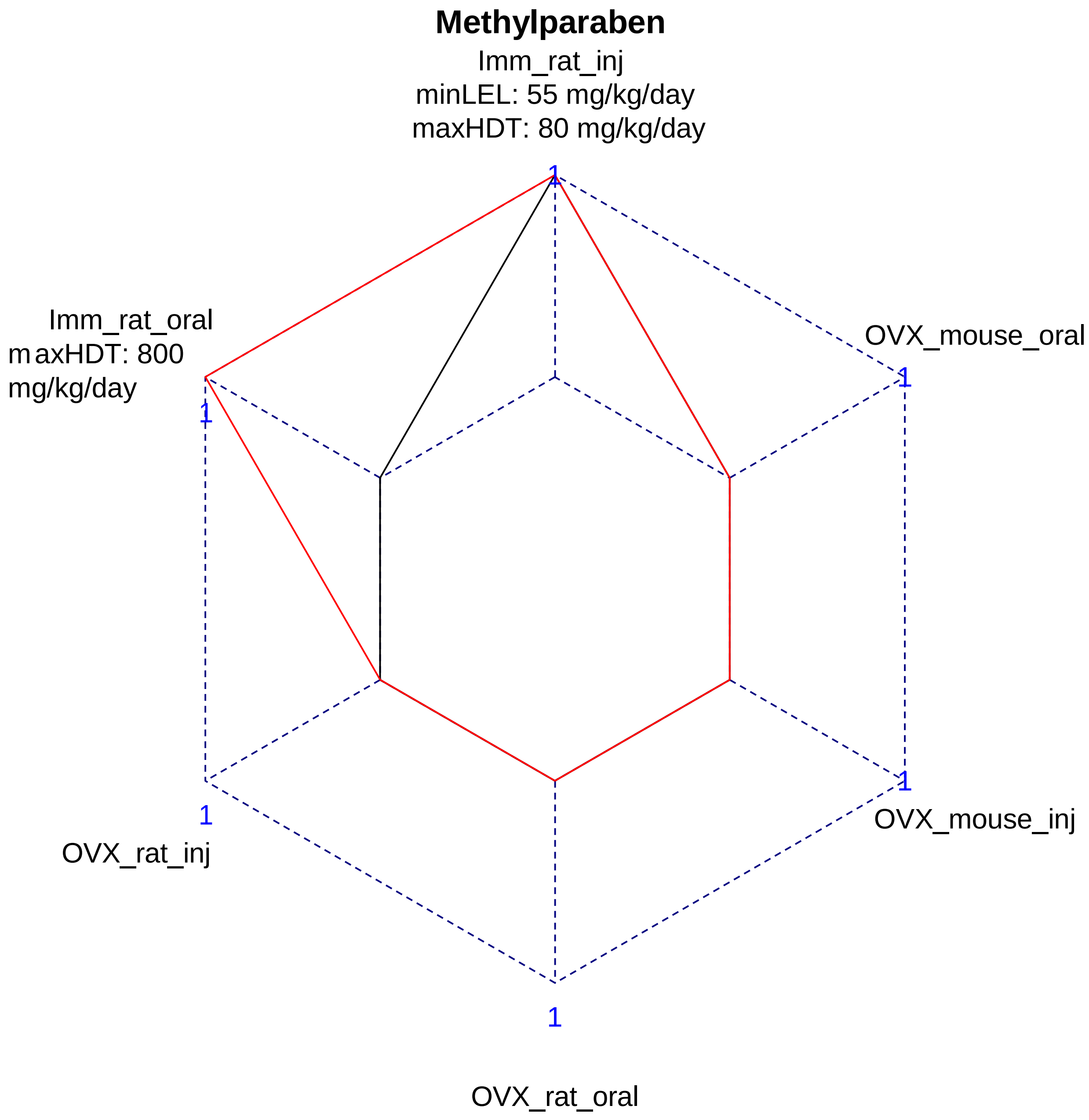
Methylparaben
Imm_rat_inj
minLEL: 55 mg/kg/day
maxHDT: 80 mg/kg/day
1
OVX_mouse_oral
1
Imm_rat_oral
maxHDT: 800
mg/kg/day
1
1
OVX_mouse_inj
1
OVX_rat_inj
1
OVX_rat_oral

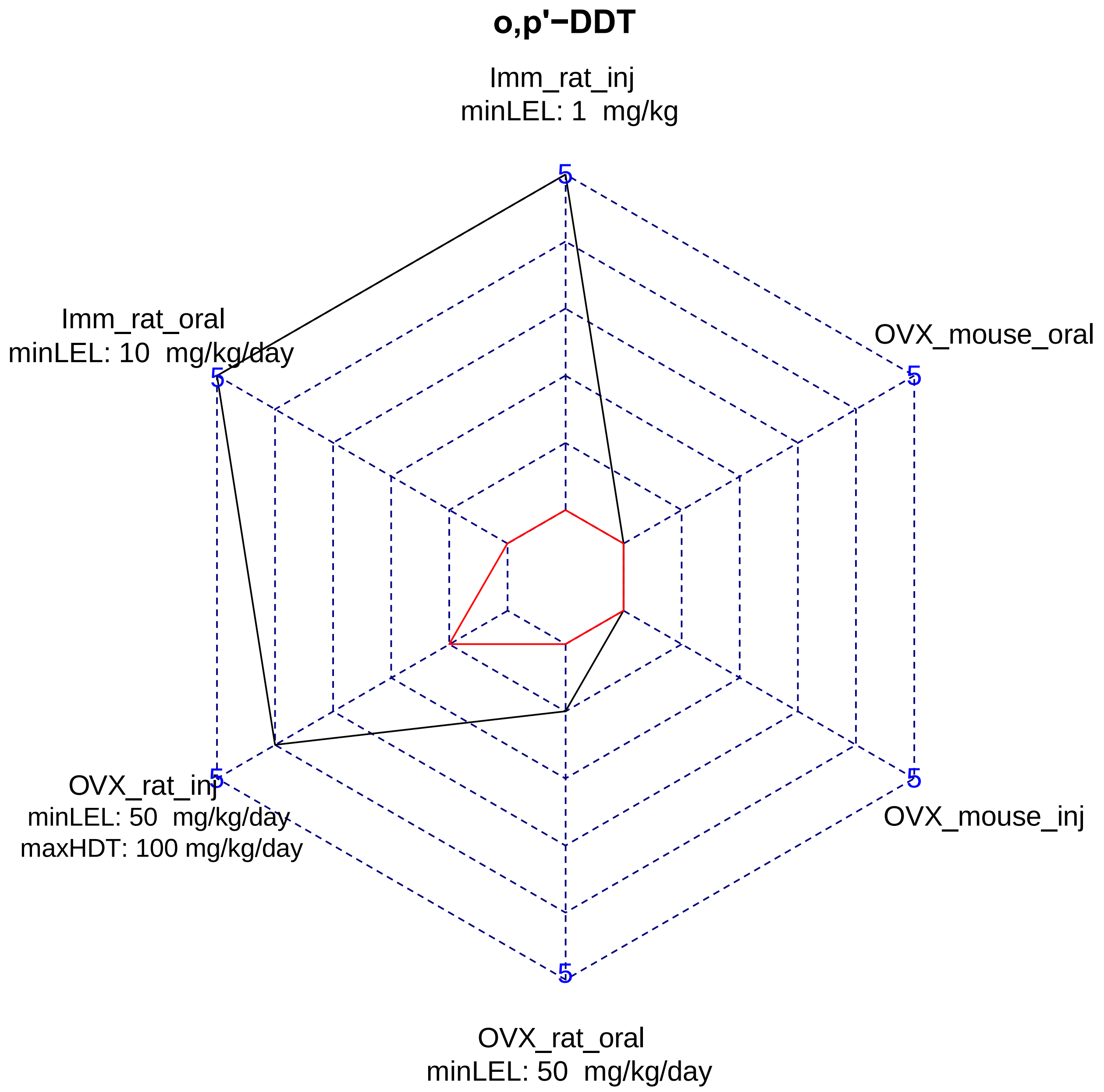
o,p'−DDT
Imm_rat_inj
minLEL: 1 mg/kg
OVX_mouse_oral
Imm_rat_oral
minLEL: 10 mg/kg/day
OVX_rat_inj
minLEL: 50 mg/kg/day
maxHDT: 100 mg/kg/day
OVX_mouse_inj
OVX_rat_oral
minLEL: 50 mg/kg/day
5
5
5
5
5
5

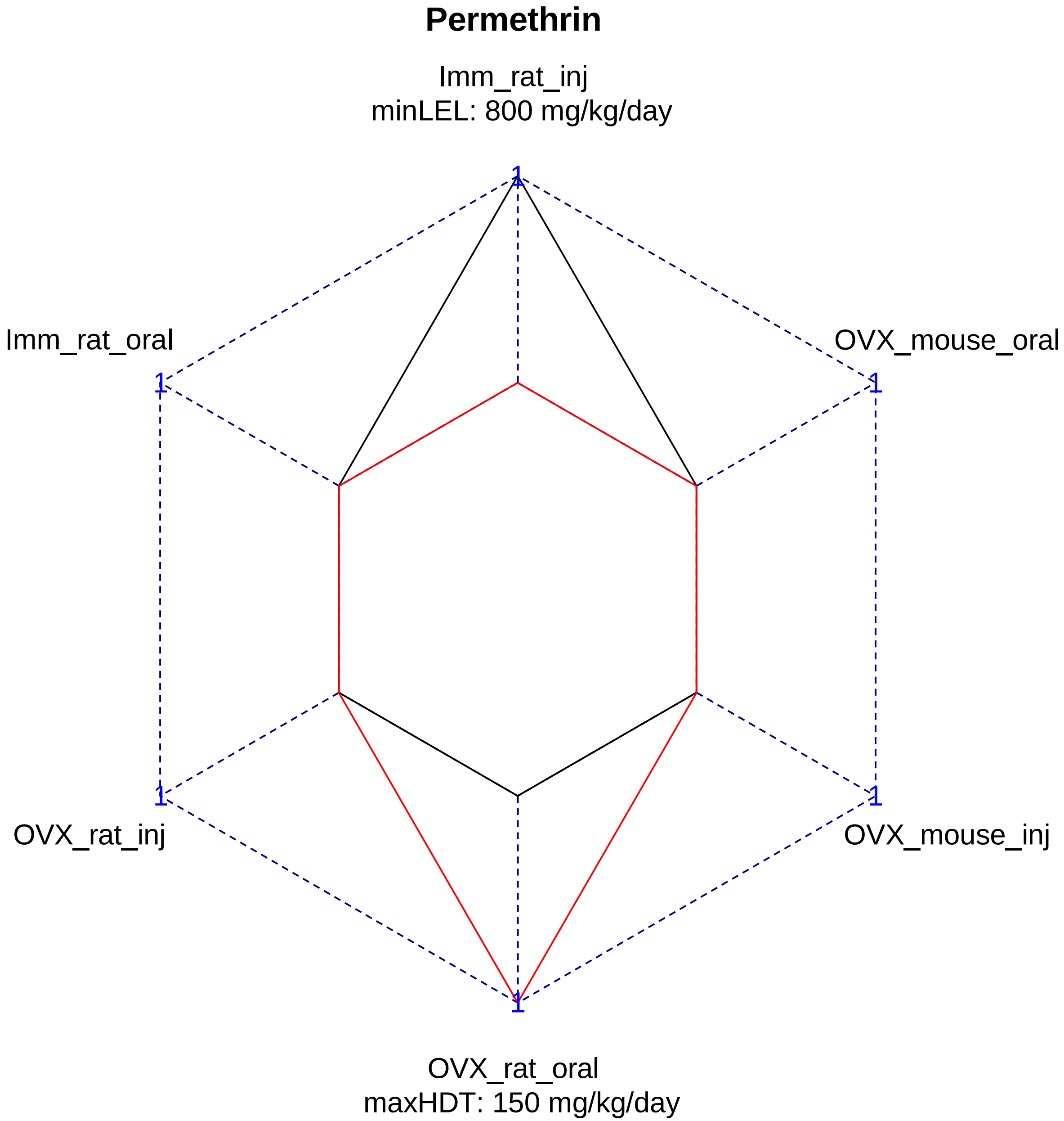
Permethrin
Imm_rat_inj
minLEL: 800 mg/kg/day
OVX_mouse_oral
OVX_mouse_inj
OVX_rat_oral
maxHDT: 150 mg/kg/day
OVX_rat_inj
Imm_rat_oral
1
1
1
1
1
1

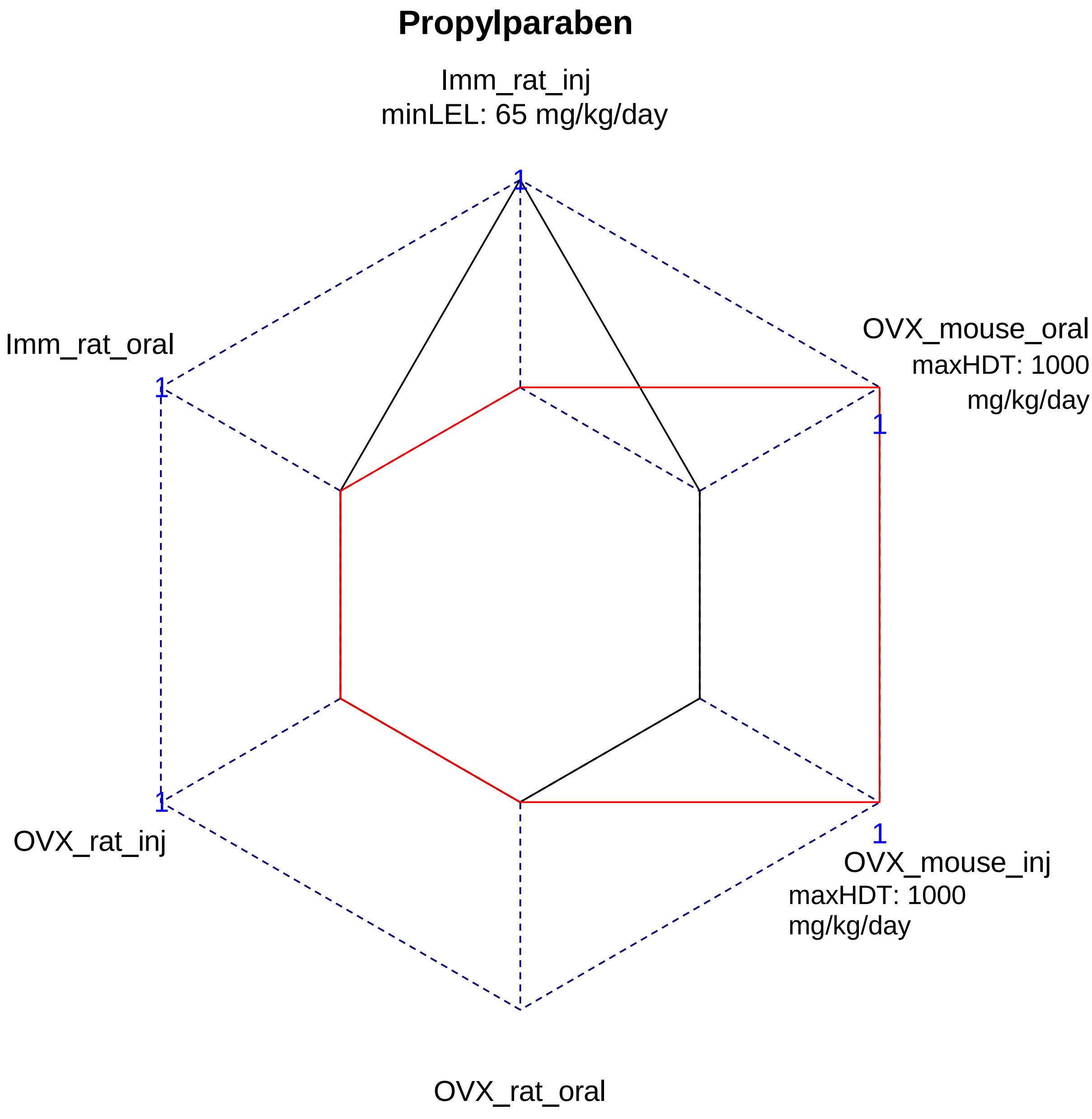
Propylparaben
Imm_rat_inj
minLEL: 65 mg/kg/day
1
OVX_mouse_oral
maxHDT: 1000
mg/kg/day
1
Imm_rat_oral
1
1
OVX_rat_inj
1
OVX_mouse_inj
maxHDT: 1000
mg/kg/day
OVX_rat_oral

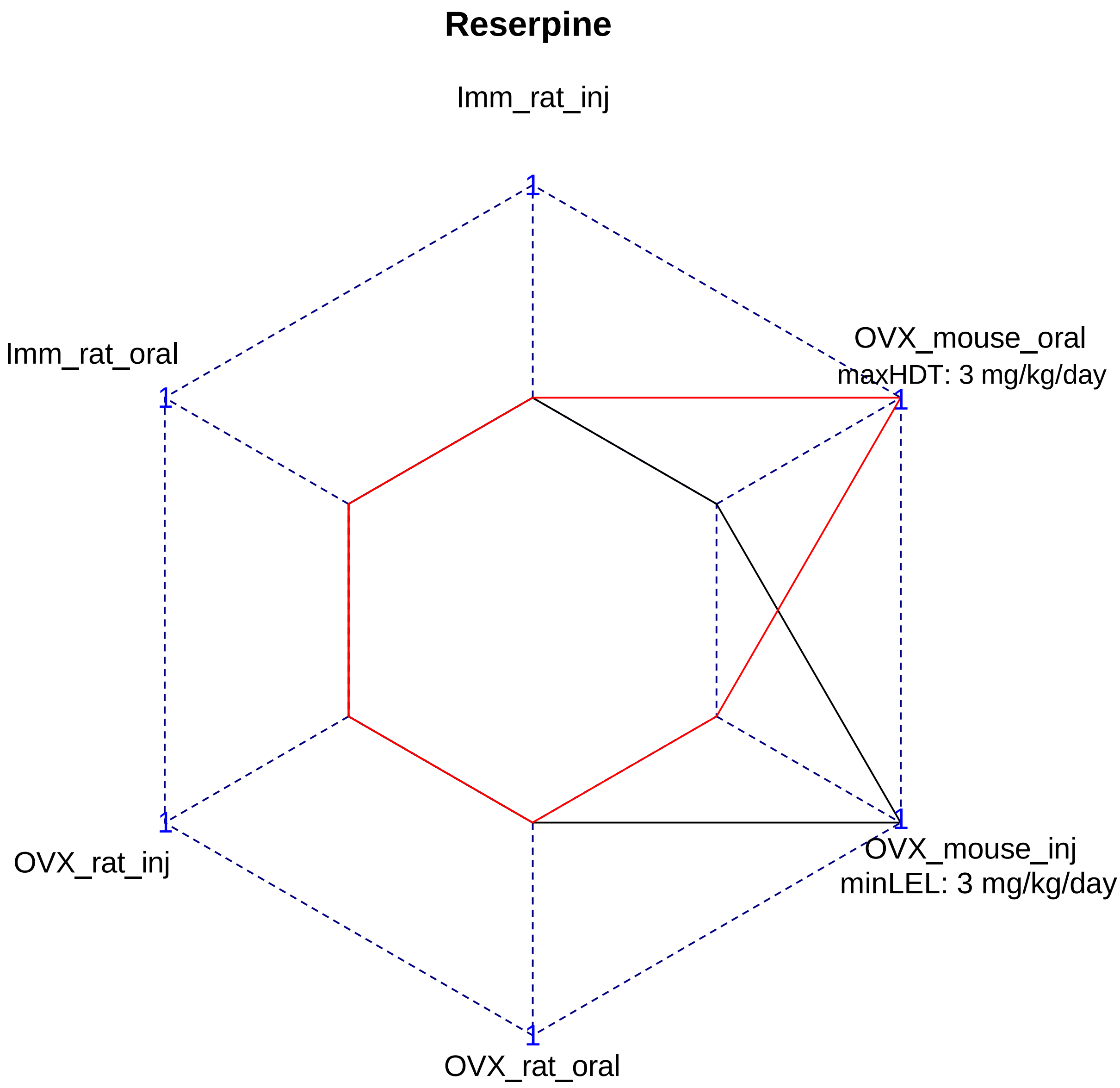
Reserpine
Imm_rat_inj
OVX_mouse_oral
maxHDT: 3 mg/kg/day
Imm_rat_oral
1
1
1
1
1
1
OVX_rat_inj
OVX_mouse_inj
minLEL: 3 mg/kg/day
OVX_rat_oral
